# Markers of Chemical and Microbiological Contamination of the Air in the Sport Centers

**DOI:** 10.3390/molecules28083560

**Published:** 2023-04-18

**Authors:** Justyna Szulc, Małgorzata Okrasa, Małgorzata Ryngajłło, Katarzyna Pielech-Przybylska, Beata Gutarowska

**Affiliations:** 1 Department of Environmental Biotechnology, Lodz University of Technology, 90-530 Łódź, Poland; 2 Department of Personal Protective Equipment, Central Institute for Labour Protection—National Research Institute, 90-133 Łódź, Poland; 3 Institute of Molecular and Industrial Biotechnology, Lodz University of Technology, 90-573 Łódź, Poland; 4 Institute of Fermentation Technology and Microbiology, Lodz University of Technology, 90-530 Łódź, Poland

**Keywords:** air contamination, VOCs, particulate matter, CO_2_, bioaerosol, sport center

## Abstract

This study aimed to assess the markers of chemical and microbiological contamination of the air at sport centers (e.g., the fitness center in Poland) including the determination of particulate matter, CO_2_, formaldehyde (DustTrak™ DRX Aerosol Monitor; Multi-functional Air Quality Detector), volatile organic compound (VOC) concentration (headspace solid-phase microextraction coupled with gas chromatography–mass spectrometry), the number of microorganisms in the air (culture methods), and microbial biodiversity (high-throughput sequencing on the Illumina platform). Additionally the number of microorganisms and the presence of SARS-CoV-2 (PCR) on the surfaces was determined. Total particle concentration varied between 0.0445 mg m^−3^ and 0.0841 mg m^−3^ with the dominance (99.65–99.99%) of the PM_2.5_ fraction. The CO_2_ concentration ranged from 800 ppm to 2198 ppm, while the formaldehyde concentration was from 0.005 mg/m^3^ to 0.049 mg m^−3^. A total of 84 VOCs were identified in the air collected from the gym. Phenol, D-limonene, toluene, and 2-ethyl-1-hexanol dominated in the air at the tested facilities. The average daily number of bacteria was 7.17 × 10^2^ CFU m^−3^–1.68 × 10^3^ CFU m^−3^, while the number of fungi was 3.03 × 10^3^ CFU m^−3^–7.34 × 10^3^ CFU m^−3^. In total, 422 genera of bacteria and 408 genera of fungi representing 21 and 11 phyla, respectively, were detected in the gym. The most abundant bacteria and fungi (>1%) that belonged to the second and third groups of health hazards were: *Escherichia-Shigella*, *Corynebacterium*, *Bacillus*, *Staphylococcus*, *Cladosporium*, *Aspergillus*, and *Penicillium*. In addition, other species that may be allergenic (*Epicoccum*) or infectious (*Acinetobacter*, *Sphingomonas*, *Sporobolomyces*) were present in the air. Moreover, the SARS-CoV-2 virus was detected on surfaces in the gym. The monitoring proposal for the assessment of the air quality at a sport center includes the following markers: total particle concentration with the PM_2.5_ fraction, CO_2_ concentration, VOCs (phenol, toluene, and 2-ethyl-1-hexanol), and the number of bacteria and fungi.

## 1. Introduction

In the modern world, great attention is paid to a healthy lifestyle that includes regular sporting activities that contribute to maintaining a healthy body weight, feeling good, and sustaining energy and a youthful appearance [1,2]. Physical activity can also prevent hypertension and non-communicable diseases (e.g., heart disease, stroke, diabetes, and site-specific cancers) [3].

According to the World Health Organization (WHO) guidelines, adults need at least 2.5 h of moderate-intensity physical activity weekly [3]. The Deloitte report “Sports Retail Study 2020” mentions that almost 65% of Europeans practice at least one sport discipline, devoting 8.6 h a week to physical activity [4]. Although physical activity has been documented as beneficial to human health, using sports facilities has raised concerns during the COVID-19 pandemic as contributing to the spread of SARS-CoV-2.

Various factors influence air quality in sports facilities (e.g., building construction, materials used, ventilation, air humidity and temperature, number of users, and type of physical activity) [5,6]. Many of these factors can favor the spread and multiplication of microorganisms (i.e., high air humidity from the intense sweat discharge of the users, high particulate matter concentration from the resuspension of particles sedimented on the surfaces, and regular contact between the users and sports equipment) [1].

Most of the air is inhaled through the mouth during physical activities excluding the normal nasal mechanisms for filtration. The increased airflow velocity carries airborne contaminants deeper into the respiratory tract. Thus, increased concentrations of microorganisms, their fragments and metabolites can be introduced into the respiratory tract of exercising individuals and pose a considerable health risk to them [1]. Moreover, the research shows that physical activity increases aerosol emissions due to elevated ventilation and dehydration of the airways, further elevating the bioaerosol concentrations. Furthermore, the air quality in sports facilities depends on CO_2_ and other gases and volatile organic compound (VOC) concentrations [7]. With the increase in the intensity of physical activity (and thus breathing), the concentration of CO_2_ in sports halls increases. According to the American Society of Heating, Refrigerating, and Air-Conditioning Engineers (ASHRAE), the maximum level of CO_2_ in sports facilities is 1000 ppm. Higher CO_2_ concentrations indicate poor air quality and acute health symptoms in room users (e.g., headaches and irritation of mucous membranes) and slower work efficiency [8]. Volatile organic compounds (VOCs) are gaseous and can originate from building materials and equipment (furniture, installations, electronics) [8,9].

Moreover, they are also associated with cleaning, disinfection, and using chemicals and cosmetics. Monocyclic aromatic hydrocarbons (MAH) are particularly important in the VOC group. VOCs can cause serious health effects as many of them exhibit toxic, carcinogenic, mutagenic, or neurotoxic properties. Many VOCs are odorous [8,9].

Bioaerosols are one of the main transmission routes for infectious diseases [10]. Moreover, human exposure to bioaerosols is associated with a wide range of acute and chronic health problems such as asthma, hay fever, bronchitis, chronic lung failure, diseases of the cardiovascular system, catarrh of the gastrointestinal tract, tuberculosis, legionellosis and allergic reactions as well as sinus and conjunctivitis [10,11]. Toxins of microbial origin (endotoxins and mycotoxins) play a significant role in inflammatory responses and contribute to the deterioration of lung function, causing other infections [12]. It has been found that over 80 types of fungi (mostly belonging to *Cladosporium*, *Alternaria*, *Aspergillus*, and *Fusarium* genera) can cause respiratory allergy symptoms and over 100 severe human and animal infections as well as plant diseases [13].

Because humans carry 1012 microorganisms in their epidermis and 1014 microorganisms in the digestive tract, they can pose as the primary source of microorganisms in fitness facilities [14]. Therefore, surfaces in sports facilities can also be a source of pathogenic microorganisms such as methicillin-resistant *Staphylococcus aureus* (MRSA). It was found that skin-to-skin contact is a primary route of MRSA transmission between athletes, especially in football, wrestling, rugby, and soccer players. Moreover, poor hygiene in equipment has also been implicated in the spread of contagious diseases [15]. Infections caused by MRSA are often aggressive, necrotizing, antibiotic-resistant, and sometimes fatal [16]. Sports facilities have already been the subject of microbiological research. The literature indicates different microbiological air contamination of these types of facilities, ranging from 5.80 × 10^1^ to 1.02 × 10^3^ CFU m^−3^ for the number of bacteria and from 2.10 × 10^1^ to 1.44 × 10^2^ CFU m^−3^ for the number of fungi [1,5,17,18]. Conversely, in the case of the bacterial contamination of surfaces, concentrations from 3.9 × 10^2^ CFU cm^−2^ to 3.7 × 10^3^ CFU cm^−2^ were observed [5,7]. Bacteria of the genera *Bacillus*, *Corynebacterium*, *Kocuria*, *Micrococcus*, and *Pseudomonas Staphylococcus* were characteristic of bioaerosols in sports facilities, while on surfaces, the dominance of *Staphylococcus*, *Bacillus*, *Klebsiella*, *Escherichia*, *Enterococcus*, *Serratia*, *Aerococcus*, and *Erwinia* has been described [19,20]. The environment of fitness centers, however, has not yet been comprehensively investigated in terms of the number and species of microorganisms present in these places.

Therefore, this study aimed to assess the markers of chemical and microbiological contamination of the air in sports centers. The research included the evaluation of microclimate parameters, particulate matter concentration, selected chemical contaminations, the number of microorganisms in the air and on surfaces, the diversity of microorganisms, and the presence of SARS-CoV-2 in fitness center environments. This is the first study to assess the biodiversity of sports facilities using the metagenome analysis of settled dust. The results are discussed in the context of pathogen transmission and the overall health effects of exposure to the detected contaminants. Moreover, guidelines for maintaining good air quality in sports facilities are proposed.

## 2. Results and Discussion

### 2.1. Microclimate and Particulate Matter Concentration

The mean values of microclimatic conditions and PM concentrations are presented in Table 1. The microclimate parameters were also analyzed as daily averages (Figure S1a). The measurements were also taken depending on the time of day (Figure S1b) and the sampling location (Figure S1c).

Airflow velocity measured during the experiments ranged from 0 m s^−1^ (no ventilation or air conditioning, no windows open, minimal number of people present at the sampling site) to 0.69 m s^−1^ (near an open window). The temperature was between 12.8 °C and 29.8 °C, and the relative humidity was between 44.3% and 78.3%. Microclimatic conditions are essential for achieving the optimal performance and comfort during exercise. The literature shows that an effective temperature below 22 °C degrades exercise performance among women, while an air temperature of 24 °C, with moderate RH, low air velocity, and weak radiation, is recommended at gyms to support exercise, comfort, and energy conservation [21]. The International Fitness Association sets different recommendations for temperature and humidity at commercial gyms [22]. A room temperature below 20 °C degrees and 50% humidity are recommended for aerobic classes, while for aerobics, cardio, weight training and Pilates areas, temperatures should be between 18 and 20 °C with a humidity between 40% and 60%.

The relation between thermal comfort and air movement at elevated activity levels was also investigated [23]. Air movement with higher temperatures produced equal or better comfort and perceived air quality below the reference condition for every temperature up to 26 °C. In our study, the air velocities and temperatures did not differ daily; the only difference was observed in the average daily humidity between Friday and the rest of the week (Figure S1a). Moreover, the air velocities did not depend on the time of day; the measurement was carried out while the temperature rose by the hour and the relative humidity first dropped (while the air conditioning was running) and then increased (Figure S1b).

The temperature conditions at different locations were similar (no statistical differences in average temperature were detected; Figure S1c). Statistically significant differences were detected for the average air velocity and average humidity (Figure S1c), corresponding to the number of windows open, the air conditioning running, and the number of people in the facility. The diversified values of these microclimatic parameters might lead to different development conditions for microorganisms between the tested locations.

The fitness center environment is very unstable, and many factors affect the parameters of temperature, relative humidity and airflow velocity. In the present study, significantly different air velocity and air humidity conditions were noted, which implies that the microclimate parameters strongly depend on the specific location. There was no correlation between the individual microclimate parameters and the number of windows open, air conditioning running, and the number of people in the facility.

Previous research has shown that the high number of people who exercise at closed sports facilities can contribute to the air quality issues inside them. The factors determining exercise intensity also affect air quality [24]. The concentration of fine particles in the indoor air fluctuates depending on the weather conditions. Furthermore, ventilation and air filtration systems at such facilities are essential for proper air exchange and purification. The present study confirmed these results as significantly higher PM concentrations were observed indoors than outdoors (Table 1).

The size distributions of airborne particles for each separate sampling variant are presented in Table S1 (Supplementary Materials). The PM_2.5_ constituted almost all measured dust at the tested locations. Its share in the total quantity of the measured airborne particles was between 99.65% and 99.99%, and the range of the number of particles per size dropped with an increasing particle size. The total suspended PM concentration varied between 0.0445 mg m^−3^ and 0.0841 mg m^−3^ and differed significantly between all of the tested locations (Figure S2c). The daily averages were higher in the first three days of the experiment and significantly lower in the last two (Figure S2a). Considerably higher concentrations were observed at the beginning of each day and just before the facility was closed (Figure S2b). Based on full-factorial ANOVA, the main effects and all interactions were confirmed at a significance level of 0.05.

According to EU legislation, the annual average concentration of dust with dimensions below 2.5 µm (i.e., the fraction containing the PM_1_ fraction) should not exceed 0.025 mg m^−3^ [25]. In our case, the measured PM_2.5_ concentration was more than twice as high as the environmental threshold, independent of the sampling site [25]. This agrees with the literature suggesting that the air quality inside training facilities is often worse than that outdoors [26].

The presence of airborne particulate matter can affect the users’ health and decrease their physical performance by around 5% [27,28]. Studies show that exposure to high PM concentrations can increase the risk of suffering from various respiratory and circulatory diseases [29,30]. Moreover, some studies have suggested that people who regularly exercise are more prone to experiencing the effects of air pollutants than those who do not participate in sports [31,32].

Carbon dioxide (CO_2_) is the main gaseous air pollutant in sports facilities, connected to a natural product of human respiration [8]. The CO_2_ concentration in the fitness club ranged from 800 ppm (reception desk) to 2198 ppm (gym), and for most rooms, it was statistically significantly higher than in atmospheric air. In previous studies, the CO_2_ concentrations in the air of sports halls were lower and ranged from 294.8 to 1529 ppm [8]. However, the current studies have not shown any exceedance of the CO_2_ concentration limits in the air developed by the WHO and the U.S. Environmental Protection Agency [33,34].

Formaldehyde concentration in the tested sports facilities ranged from 0.005 mg/m^3^ to 0.049 mg/m^3^, and for two out of five rooms, it was statistically significantly lower than in the control (atmospheric) air. This is probably due to the heavy traffic in the parking lot adjacent to the building and the busy street. According to EPA guidelines, the detected values of formaldehyde do not exceed the limits for this compound in the air [34].

### 2.2. Volatile Compounds Contamination

The volatile compounds were extracted from the air sample by SPME, followed by desorption and analysis with GC-MS. A total of 85 compounds were identified, of which 84 were present in the air collected from the gym, while only 47 were identified in the control sample (background) (Table S1). Detected compounds were divided into ten groups based on their chemical structures: hydrocarbons (30), terpenes and terpenoids (20), alcohols (10), aldehydes (eight), ketones (six), esters (six), furanes (two), phenols (one), ethers (one), and acids (one).

The sources of the compounds that were identified in the indoor air from the gym can differ; for example, the breath air exhaled by people in the gym [35] (e.g., acetone, ethanol, 1-propanol, butyl acetate, acetic acid, acetoin, and 2,3-butanedione), alcohol-based hand disinfectants and equipment cleaning agents [36], air fresheners and cosmetics [37] (e.g., ethanol, phenol, benzaldehyde, 2-propanol, α-pinene, eucalyptol, linalool, 3-carene, D-limonene, γ-terpinene, α-thujene, β-myrcene, camphene, butane, pentane, acetone, furfural, 3-methyl-1-butanol, 2-methyl-1-butanol, dihydromyrcenol, citronellal, verbenone, menthol), building materials and room finishing materials [38], and gym equipment (e.g., furfural, toluene, benzene, styrene, xylenes, ethylbenzene, heptane, decane, benzaldehyde, hexanal).

The relative amount (%) results showed that phenol was the dominant compound in both the control air sample and air sample from the gym. The presence of phenol results from the widespread use of it and its derivatives, among others, in the production of resins, detergents, medicinal products, disinfectants, and dyes, and thus are found in many common materials including antiseptics, medical preparations, plastics, cosmetics, and health care products [39]. Phenol also gets into the air through car exhaust. Phenol is not classified as a carcinogen but as a toxic substance [40]. Due to its hydrophilic and lipophilic properties, phenol easily penetrates cell membranes and dissolves in cell fractions, causing interaction with specific cellular and tissue structures [41].

Three other identified compounds, D-limonene, toluene, and 2-ethyl-1-hexanol, were characterized by over 1–2% of the relative amount in the indoor air from the gym. Among the indoor air VOCs, terpenes are a common group. Cleaning agents and cosmetics contain essential oils rich in terpenes [42].

Toluene, along with benzene, ethylbenzene, and xylenes from the BTEX group, are classified as toxic compounds, while benzene is also classified as a carcinogenic substance (Group 1). Due to their application for various purposes such as the production of plastics, synthetic fibers, floor coverings, chipboard, oils, greases, and paint, the presence of BTEX is common in indoor air. Long-term exposure to BTEX increases the risk of adverse health consequences [43].

In turn, 2-ethyl-1-hexanol is a common component of fragrances. Moreover, it is commonly used for the production of plasticizers (e.g., diethylhexyl phthalate for polyvinyl chloride resins) as well as in coating products, greases, fillers, and putties. The presence of 2-ethyl-1-hexanol may irritate the mucous membranes of the eyes and nose in humans [44,45].

### 2.3. Determination of Airborne Microorganism Number

**Figure 1:**
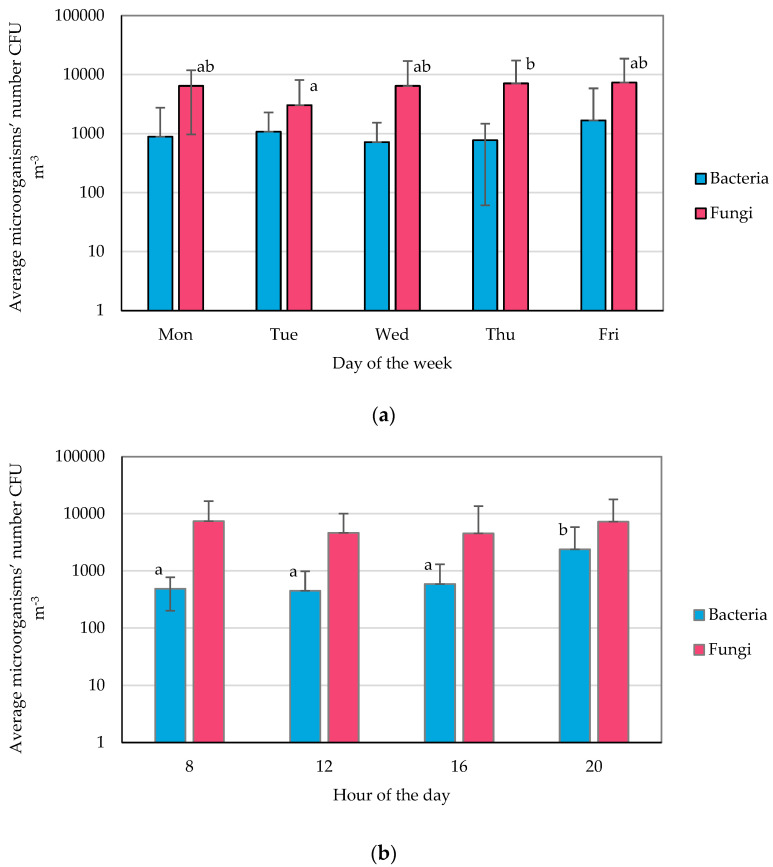

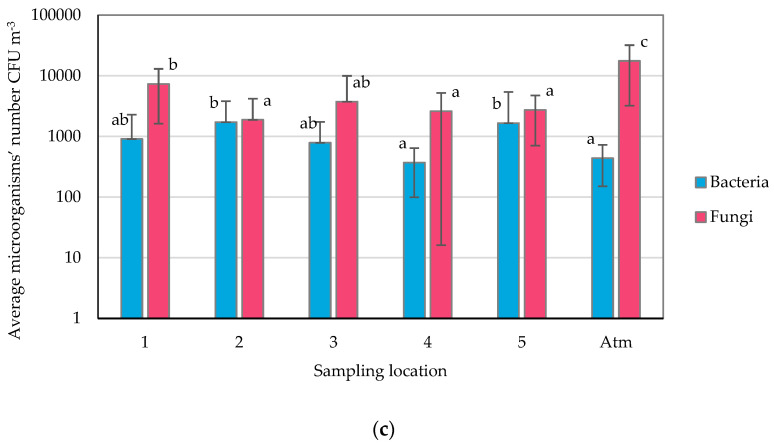
Averaged microorganism numbers (means with SD): (**a**) daily, (**b**) over sampling time of the day, (**c**) over sampling location; statistically different samples are marked with different letters; no letters indicate no statistical differences (Tukey’s test, α = 0.05).

The average daily number of bacteria in the facilities during the working week ranged from 7.17 × 10^2^ CFU m^−3^ (Wednesday) to 1.68 × 10^3^ CFU m^−3^ (Friday), while the number of fungi ranged from 3.03 × 10^3^ CFU m^−3^ (Wednesday) to 7.34 × 10^3^ CFU m^−3^ (Friday) (Table S3, Figure 1a). The lowest number of bacteria was recorded at 08:00 (4.48 × 10^2^ CFU m^−3^) and the highest at 20:00 (2.39 × 10^3^ CFU m^−3^). In turn, the concentration of fungi was the lowest at 16:00 (4.53 × 10^3^ CFU m^−3^) and the highest at 08:00 (7.42 × 10^3^ CFU m^−3^) (Table S3, Figure 1b). The most contaminated air was observed in Room no. 2 (the gym), where the bacteria count was 1.66 × 10^3^ CFU m^−3^, and in Room no. 1 (the reception), where the number of fungi was 7.30 × 10^3^ CFU m^−3^ (daily mean).

It is noteworthy that, at the same time, in Room no. 2, the lowest number of fungi (1.90 × 10^3^ CFU m^−3^) among the analyzed rooms was recorded. In turn, the lowest number of bacteria was found in Room no. 4 (fitness room on the second floor) (Table S3, Figure 1c).

No statistically significant differences were found in the fitness club’s mean daily numbers of bacteria. In the case of fungi, significant differences among days were observed, with the lowest concentration on Tuesday and the highest on Thursday (Figure 1a). Considering the influence of the test hour on the number of microorganisms in the air; it can be concluded that the number of fungi is constant while the number of bacteria changes, which is most likely related to the activity of people in these facilities. Statistically higher numbers of bacteria were recorded at the end of the day—at 20:00 (Figure 1b). It was also shown that the number of fungi in the atmospheric air was statistically significantly higher than in the samples collected at the fitness club (Figure 1c).

**Figure 2:**
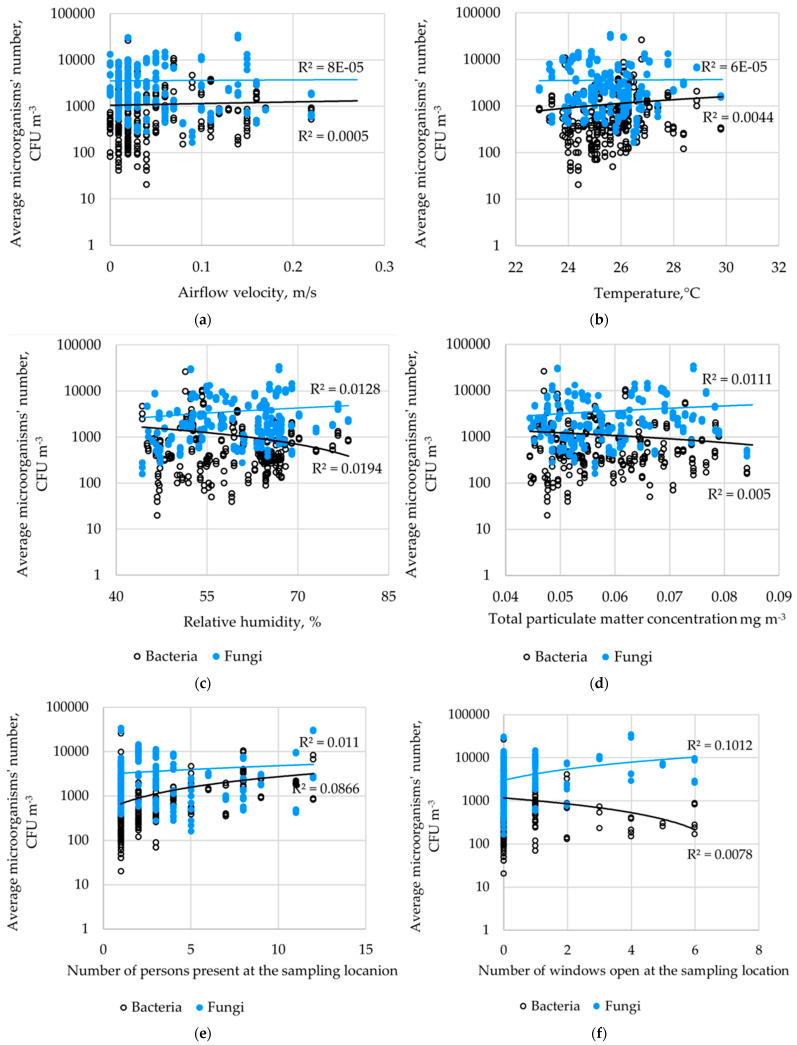
Correlation between microorganism numbers and the: (**a**) airflow velocity, (**b**) temperature, (**c**) relative humidity, (**d**) total particulate matter concentration, (**e**) the number of persons present at the sampling location, and (**f**) the number of windows open at the sampling location.

The obtained aggregate results from the week of air quality monitoring in the fitness club were subjected to detailed statistical analysis, which showed a very weak correlation between the average number of bacteria and fungi in the air, and the airflow, temperature, relative humidity, and the number of particles in the air (Figure 2a–d). Moreover, the correlation between the number of microorganisms in the air, the number of persons present at the sampling location, and the number of open windows were also very weak (Figure 2e,f).

It is worth noting that the present research showed higher microbiological air contamination (bacteria: 7.17 × 10^2^–1.68 × 10^3^ CFU m^−3^, fungi: 03 × 10^3^ CFU m^−3^–7.34 × 10^3^ CFU m^−3^) than that in previously published studies. In addition, previous research focused primarily on assessing microbial contamination in sports facilities in schools and universities. Brągoszewska et al. recorded the number of bacteria in the air in a Polish high school gym from 4.20 × 10^2^ to 8.75 × 10^2^ CFU m^−3^ depending on the activity of the students [17]. Additionally, other studies conducted in Europe (gyms, fitness rooms, and different facilities in academic sport centers) have shown lower microbial contamination (i.e., 5.80 × 10^1^ to 2.00 × 10^4^ CFU m^−3^ for the number of bacteria in the air, and 2.10 × 10^1^ to 3.75 × 10^2^ CFU m^−3^ for the number of fungi in sports facilities) [1,7].

Recently, Boonrattanakij et al. investigated microbial contamination in a bicycle room at a fitness center in Taiwan using the same type of air sampler and culture media as the current study [5]. The authors obtained a lower number of bacteria (4.01 × 10^2^–7.61 × 10^2^ CFU m^−3^) and fungi (2.26 × 10^2^–8.37 × 10^2^ CFU m^−3^).

It should be noted that many factors can be responsible for the differences in microbial contamination shown in current and previous studies, such as building construction and materials, ventilation systems and environmental factors (season, air humidity, temperature) and others [5,6].

Unfortunately, there are no legal limits on the number of microorganisms in indoor air to which the results obtained in the present study could be referred. The WHO suggests that the total number of microorganisms should not exceed 1.0 × 10^3^ CFU m^−3^. At the same time, the Polish Commission for Maximum Admissible Concentrations and Intensities for Agents Harmful to Health in the Working Environment has developed limits of 5.0 × 10^3^ CFU m^−3^ for the total number of mesophilic bacteria, and the total number of fungi for residential and public utility facilities [46].

Considering the average daily values of the number of microorganisms obtained in the present study, the number of bacteria in Rooms no. 2 and 5 was exceeded. In addition, the number of fungi in all rooms exceeded the WHO’s recommendations [47]. Referring to the Polish guidelines, only the number of fungi in Room no. 1 (reception) was exceeded, where, most often, there was an open window allowing for the inflow of atmospheric air, which was highly contaminated with fungi during the research period.

Statistical analysis showed that atmospheric air could be the fungi source in the fitness club rooms tested. This hypothesis could be tested by comparing the indoor air’s fungal composition to the outdoor air. In contrast, the source of bacteria in the indoor air was probably of human origin, as suggested by the highest values observed in the room where the most intense exercises were performed. This conclusion is supported by previous studies showing that bacteria can be over two times more abundant indoors than outdoors, especially in poorly ventilated and heavily occupied premises [14,48].

Although it is known that microclimate conditions, especially temperature and relative air humidity, as a rule, correlate with the number of microorganisms in the air in the rooms [49,50], this was not observed in the current research. This is probably because the environment of fitness clubs is very specific and unstable, which is mainly related to the varying number of people, who are carriers of specific microbiota, perform exercises of varying intensity, enter/leave rooms, open/close doors, open/close windows, turn on/off fans and air-conditioners, etc. These overlapping factors have unpredictable results; future research should introduce systems for continuously monitoring the microbiological air quality in sports facilities.

### 2.4. Determination of Surface Microbial Contamination

**Figure 3:**
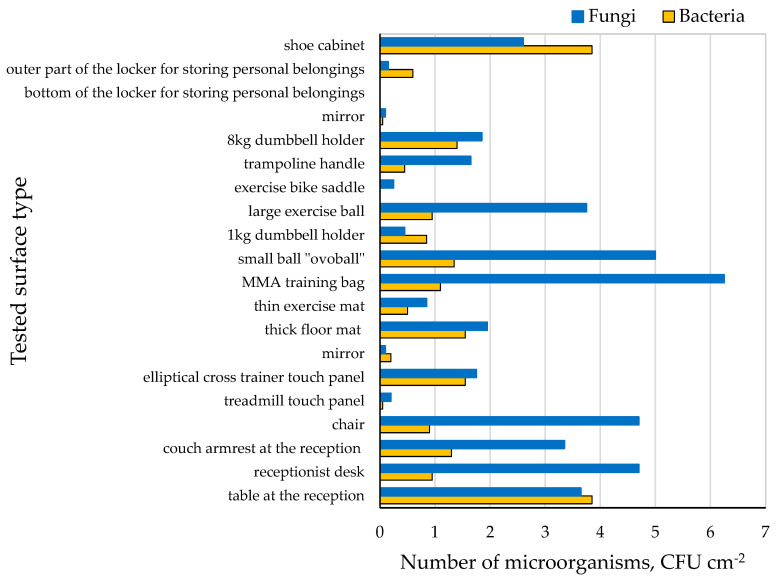
Microbiological contamination of surfaces.

The highest number of bacteria was found in the shoe cabinet and on the table in the reception area, which was used by people waiting (3.8 CFU cm^−2^). No bacteria were found on the exercise bike saddle and at the bottom of the locker used to store personal belongings (0 CFU cm^−2^). The highest number of fungi was found on the MMA training bag (6.2 CFU cm^−2^), while no fungi were present at the bottom of the storage locker (Figure 3). Significant differences were observed in the concentration of bacteria and fungi between the tested surfaces (*p* < 0.05).

Few studies have presented a quantitative assessment of microbial surface contamination in sports facilities. In the present study, surface microbiological contamination was lower than that in the previously published studies. Boonrattanakij et al. conducted microbiological tests on sports equipment (i.e., bicycle handle, dumbbell, and sit-up bench) [5]. The number of bacteria on the examined surfaces (bicycle handle, dumbbell, and sit-up bench) ranged from 3.9 × 102 CFU cm^−2^ to 3.7 × 10^3^ CFU cm^−2^.

Notably, guidance was posted in the cloakroom and gym for users to disinfect the exercise equipment and cabinets for personal belongings to prevent the spread of COVID-19. Based on the obtained results, it can be concluded that the users did not follow the recommendations in all cases and/or disinfection was ineffective.

### 2.5. Diversity of Microorganisms in the Fitness Center Environment

**Figure 4:**
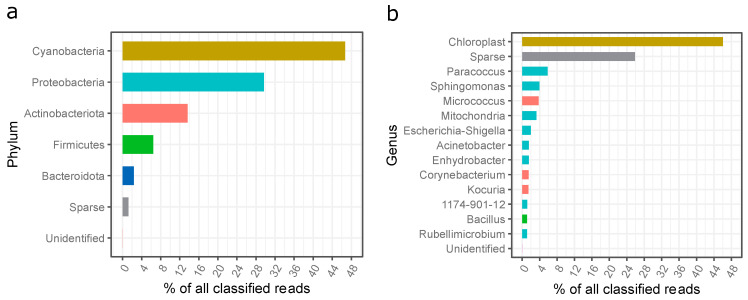
Phylogenetic distribution of bacteria sequences in the settled dust sample; (**a**) assigned to the phyla, (**b**) assigned to the genera; unidentified—unidentified sequences; sparse—reads assigned to low abundant phyla (less than 1% of all classified reads).

The results from the high-throughput DNA sequencing of the settled dust sample collected at the fitness club revealed a high diversity of microorganisms (Figure 4a). In total, four hundred and twenty-two (422) genera of bacteria representing 21 phyla were detected in the dust. Although the number of phyla was high, most shared a minimal number of classified reads. The most abundant phyla belonged to Cyanobacteria (46%), Proteobacteria (30%), Actinobacteriota (14%), Firmicutes (6%), and Bacteroidota (2%) (Figure 4a). The high number of reads from the Cyanobacteria phylum was a surprise. A closer analysis of the reads based on a sequence similarity search employing the NCBI Nucleotide collection database revealed that these sequences were mainly derived from pine (*Pinus* spp.) chloroplast DNA, which suggests that the dust sample was primarily contaminated with pollen.

The most abundant bacteria identified in the settled dust from the gym in question belonged to the genus *Paracoccus* (5.8%), *Sphingomonas* (3.9%), *Micrococcus* (3.8%), *Escherichia-Shigella* (2%), *Acinetobacter* (1.5%), *Enhydrobacter* (1.5%), *Corynebacterium* (1.5%), *Kocuria* (1.5%), 1174-901-12 (*Rhizobiales*; 1.2%), *Bacillus* (1.1%), and *Rubellimicrobium* (1.1%). The presence of mitochondrial DNA was most probably due to the contamination of the dust sample with pine pollen.

Following this, the presence of potentially hazardous bacterial genera sequences, according to Directive 2019/1833/EC [51], was checked among the classified reads of the dust sample. Twenty-eight hazardous genera (Groups 2 or 3) were identified; however, their share in the total number was very low (<7.5% of all classified reads). Of these, the most abundant genera were *Escherichia-Shigella* (2%), *Corynebacterium* (1.4%), *Bacillus* (1%), and *Staphylococcus* (0.8%) (Figure 4 and Figure S3).

So far, the bacteria *Bacillus*, *Corynebacterium*, *Kocuria*, *Micrococcus*, and *Pseudomonas Staphylococcus* have been reported as characteristic of the environment of sports facilities, identified by classical (culture) methods [1,17,52]. Turkskani et al. isolated bacteria from two Saudi Arabian gyms and identified them based on gene sequences of their 16S rRNA [53]. The authors determined the phylogenetic affiliation of the detected bacteria to the following genera: *Bacillus*, *Brachybacterium*, *Geobacillus*, *Microbacterium*, *Micrococcus*, and *Staphylococcus*. Moreover, Haghverdian et al. demonstrated the prevalence and transmissibility of *S. aureus* on the surfaces (floor, balls, hands) in sports facilities [15]. The authors observed the viability of *S. aureus* on sequestered sports balls for 72 h, while another work demonstrated the survival of *S. aureus* strains for up to 12 days on inanimate surfaces [54]. Recently, Szulc et al., (2023) published the results of the first metagenomic analysis of a bioaerosol from a sports center (a room with a climbing wall). The authors identified bacteria mainly belonging to the genus *Cellulosimicrobium*, *Stenotrophomonas*, *Acinetobacter*, *Escherichia*, and *Lactobacillus* in these environments [7].

The present study detected bacteria of the *Paracoccus*, *Sphingomonas*, *Enhydrobacter*, *Rubellimicrobium* and 1174-901-12 genus, with a share of more than 15%, which have never previously been identified in sports facilities.

*Paracoccus* was isolated from various environments including soils, salines, marine sediments, wastewater, and biofilters. Most include saprophytes, but one species of *P. yeei* is known to be associated with opportunistic infections in humans [55,56]. Additionally, *Rubellimicrobium* are environmental bacteria observed in the soil, air, and slime on industrial machines [57]; therefore, their presence in a fitness club is unsurprising.

*Sphingomonas* has also been isolated from many environmental samples (soil, sediment, water) including samples chemically contaminated with azo dyes, phenols, dibenzofurans, insecticides, and herbicides [58]. Many *Sphingomonas* strains have been isolated from human clinical specimens and hospital environments where *Sphingomonas paucimobilis*, *S. mucosissima*, and *S. adhesiva* are most associated with human infections [59].

Genus 1174-901-12 has previously been isolated from soil, ceramic roofs, and photovoltaic panels [60], indicating that its source may be the external environment or building materials in the fitness club building.

So far, only one species of *Enhydrobacter* is known (*E. aerosaccus*), which was isolated from a eutrophic lake. These bacteria are rare and poorly described in the literature; therefore, it is challenging to conclude their source in the studied fitness club and their potential effects [61].

**Figure 5:**
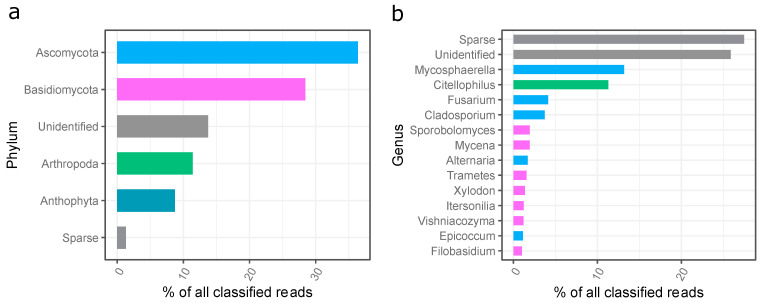
Phylogenetic distribution of fungi sequences in the settled dust sample; (**a**) assigned to the phyla, (**b**) assigned to the genera; unidentified—unidentified sequences; sparse—reads assigned to low abundant phyla (less than 1% of all classified reads).

The ITS-based analysis revealed that, in total, four hundred and eight (408) genera of fungi representing 11 phyla were detected in the dust. The most abundant phyla belonged to *Ascomycota* (36.4%), *Basidiomycota* (28.4%), *Arthropoda* (11.4%), and *Anthophyta* (8.7%) (Figure 5a).

The most abundant fungi identified in the settled dust from the gym in question belonged to the genus *Mycosphaerella* (13.2%), *Citellophilus* (11.3%), *Fusarium* (4.1%), *Cladosporium* (3.7%), *Sporobolomyces* (1.9%), *Mycena* (1.9%), *Alternaria* (1.7%), *Trametes* (1.6%), *Xylodon* (1.4%), *Itersonilia* (1.2%), *Vishaniacozyma* (1.2%), *Epicoccum* (1.1%), and *Filobasidium* (1.0%) (Figure 5b).

Seven genera of the hazardous category (Groups 2 or 3), according to Directive 2019/1833/EC were found, and their share in the total number was very low (less than 1.5% of all classified reads). Of these, the most abundant genera were *Cladosporium* (3.7%), *Aspergillus* (0.9%), and *Penicillium* (0.4%) (Figure S4).

Żyrek et al. indicated the presence of the yeast *Candida* sp. Małecka-Adamowicz et al. found fungi from the genus *Cladosporium*, and to a lesser extent, *Penicillium*, *Fusarium*, *Acremonium*, *Alternaria*, and *Aureobasidium* [1,52]. The occurrence of potentially allergenic molds of the genera *Aspergillus* and *Cladosporium* in Czech sports facilities was described in [18]. Viegas et al. identified 25 species of fungi occurring in ten gymnasia and found mainly molds from the following genera: *Cladosporium*, *Penicillium*, *Aspergillus*, *Mucor*, *Phoma* and *Chrysonilia* as well as yeasts from the genera: *Rhodotorula*, *Trichosporon mucoides* and *Cryptococcus uniguttulattus* [62]. Szulc et al. indicated the dominance of fungi: *Mycosphaerella*, *Botrytis*, *Chalastospora*, *Cladosporium*, *Itersonilia*, *Malassezia*, *Naganishia*, *Saccharomyces*, *Sporobolomyces*, *Trichosporon*, and *Udeniomyces* in sports facilities for climbing activities [7].

The results obtained in the present study differed from the literature data. The genera of fungi: *Acremonium*, *Aureobasidium*, *Penicillium*, *Aspergillus*, *Candida*, *Mucor*, *Phoma*, *Chrysonilia*, *Rhodotorula*, *Trichosporon* and *Cryptococcus*, which dominated in earlier studies, were found in low quantities, from 0.01% to 0.8% [52,62], which may be related to the seasonal variability of the types of fungi dominant in the atmospheric air shaping the qualitative composition of indoor fungi.

Moreover, in the present research, the following fungi: *Citellophilus*, *Mycena*, *Tramates*, *Xylodon*, *Vishniacozyma*, *Epicoccum*, and *Filobasidium* were identified for the first time in gym facilities. These fungi are common genera and likely come from the outdoor air. They are known as plant parasites but can also be allergic to humans, are often linked to decreased pulmonary function and asthma admissions, and may cause infections, particularly in immunosuppressed patients [63,64,65,66,67,68,69,70].

It should be mentioned that the use of high-throughput sequencing on the Illumina platform made it possible to identify a greater variety of microorganisms found in sports facilities than that previously described in the literature. Metagenomic analysis is increasingly used to study various environmental samples such as soil, water, technical materials (e.g., cardboard, cellulosic materials, collagen), settled dust, and many others [71,72,73]. The advantage of this method is the identification of microorganisms directly from the test sample, skipping the cultivation stage, which prevents the loss of species of microorganisms that cannot grow under laboratory conditions [74].

### 2.6. Assessment of SARS-CoV-2 Virus Presence in the Fitness Center Environment

Selected surfaces in Room no. 2 (gym) were tested for the SARS-CoV-2 virus. In the case of the treadmill touch panel, the result was positive (Table 2). The results suggest a real risk of the spread of the COVID-19 pandemic in gyms and fitness clubs.

It is worth mentioning that between 74 and 164 cases of COVID-19 per day were recorded in Poland between 26 and 30 of July 2021. In the province where the fitness center in question was located, cases ranged between 1 and 13 per day [75]. The detection of the SARS-CoV-2 virus suggests a real risk of the spread of COVID-19 in gyms and fitness clubs. However, studies involving a larger number of tested samples are needed to confirm this hypothesis.

The risk of the transmission of COVID-19 may arise from close contact, the emission of droplets, or through fomites. Intensive physical activities in a fitness center favor these factors, mainly due to the increased physical contact, increased concentration of exhaled respiratory droplets in a confined space because of vigorous breathing, and shared communal space and equipment [76]. No RNA of SARS-CoV-2 was detected in the previously performed air and surface studies at a fitness center in the U.S. [77].

SARS-CoV-2 transmission in sports facilities has been previously proven in positive PCR tests of infected users and workers [78]. Conversely, in Norway, Helsingen et al. tested 3764 individuals divided into two groups (with and without access to training at a fitness center) [79]. They found a difference of 0.05% (one versus zero cases) in SARS-CoV-2 RNA test positivity between training and non-training individuals. The authors stated that good hygiene and physical distance in fitness centers did not increase the infection risk of SARS-CoV-2 for individuals without COVID-19-relevant comorbidities in such spaces. Therefore, it is essential to make the users and employees of these facilities aware of the principles of sanitary safety and the proper disinfection of hands and sports equipment.

### 2.7. Directions for Minimizing Microbiological and Chemical Threats in the Sports Facilities

The benefits of physical activities should be strengthened by reducing the exposure to physicochemical and microbiological contamination and consequently by minimizing the risk of possible adverse health effects for users in sports facilities [80].

In the tested fitness club, we found a high concentration of dust, microorganisms, and the SARS-CoV-2 virus. It is worth mentioning that the performed studies had some limitations resulting from (a) the uniqueness of the sample (only one fitness club was tested); (b) the season in which the samples were taken due to the influence of external bioaerosols on the amount and composition of internal bioaerosols; (c) the holiday/vacation season, which meant that the number of users was lower than the rest of the year; (d) the small number of samples taken for analysis for the detection of SARS-CoV-2 and metagenomic analysis. However, studies suggest that air purification systems with proven effectiveness are needed for continuous operation during opening hours in sports facilities.

Various chemical and physical methods are currently known and tested for air disinfection. Air disinfection includes filtration, ozonation, exposure to ultraviolet radiation, photocatalysis, and cold plasma [81]. Recently, it has been proposed to use strong electric fields in which the destruction or electroporation of microorganisms occurs [82]. Among these disinfection techniques, chemical fogging, ozonation, and UV radiation of the air are the main solutions available on the market [81]. These methods are currently used in clinical and pharmaceutical objects; however, they seem suitable for sports facilities.

One of the ways to prevent the spread of viruses and pathogenic microorganisms in sports facilities is to use floors that feature antibacterial properties and other materials (e.g., clothing and towels) with biostatic properties.

It is worth noting that one of the practices related to preventing the spread of the COVID-19 pandemic was the introduction of spray bottles filled with a disinfectant solution in sports centers for wiping the exercise equipment after use. It should be noted that these practices have their weaknesses.

Rarely are surface disinfectants at sports facilities in their original packaging, allowing the center to control their composition and the concentration of active substances. Therefore, it is crucial to use EPA-approved disinfectants, consider the type of disinfected surfaces (metal, plastic, leather, etc.), and prepare working solutions of disinfectants following the manufacturer’s guidelines, labeling them properly, and providing detailed instructions for use for the end users. This is important because the effectiveness of disinfection will depend on the contact time of the preparation used with the surface. Common misconduct is spraying and immediately wiping off sports equipment. Such disinfection will not be effective and will even become dangerous for the user and the environment. Therefore, the staff at sports clubs must be properly prepared (trained) to use appropriate safety procedures and personal protective equipment (if necessary) during disinfection. An alternative to sprayed disinfectants can be disinfectant-impregnated wipes, consisting of towels saturated with diluted disinfectant and other compounds (i.e., surfactants, preservatives, enzymes, and perfumes) [83]. Staff and the users of exercise facilities should wash their hands with water and plain soap before entering and leaving and before and after any contact with other people and equipment in sports facilities and avoid sharing towels (preferably use disposable paper towels) and other personal items. Wounds, cuts, scrapes, etc., should be covered with a clean, dry dressing to prevent contamination. The World Health Organization (WHO) recommends alcohol-based formulations to disinfect hands; such formulations have been shown to inactivate SARS-CoV-2 efficiently.

Moreover, hydrogen peroxide or povidone-iodine and other biocides possess antiviral properties and can be used to disinfect biological surfaces [84]. The sharing of exercise equipment should be avoided if possible. If this is not possible, the use of a towel is recommended, or, for example, gloves that provide a barrier between the skin and such equipment. After the entire working day, the facility staff should wash and disinfect all common exercise equipment used on a given day. Moreover, objects inside a sports facility that require special attention include countertops, light switches, faucet handles, and doorknobs. Staff should be excluded from the use of damaged equipment (e.g., torn upholstery) that cannot be properly disinfected due to damage.

Future research should aim at introducing Internet of Things (IoT) technology systems of constant air quality monitoring in sports facilities (e.g., using multiple sensors including microfluidic chips as well as developing warning systems against exceeding the concentration of suspended dust, or the recommended number of microorganisms in the air).

## 3. Materials and Methods

### 3.1. Tested Fitness Center and Sampling Strategy

The research was conducted in a fitness club in Zduńska Wola (central Poland). The tested fitness center is located in a service and commercial building built in the1990s and operates from Monday to Friday from 8:00 to 22:00 and on the weekends from 9:00 to 15:00. The characteristics of the rooms under study are presented in Table 3. Samples of the air were collected from five fitness center rooms equipped with an occasional air conditioning system.

Moreover, control samples (atmospheric air) in front of the building were collected simultaneously. Samples were collected during the entire working week (Monday–Friday) at 8:00, 12:00, 16:00 and 20:00 under normal operating conditions. At the same time, the microclimate and particulate matter concentrations were analyzed. The microbial contamination was also assessed for 20 surfaces in the fitness center (Table 3). The chemical contamination of the air was checked in the gym (Room no. 2) in comparison to the control atmospheric air (Room no. 6).

Additionally, three samples were taken from the surface of Room no. 2 (gym) to verify the presence of the SARS-CoV-2 virus. A pooled sample of settled dust was also collected to determine the biodiversity of the microorganisms.

### 3.2. Microclimate, Particulate Matter Concentration Carbon Dioxide, and Formaldehyde Analysis

A VelociCalc^®^ Multi-Function Velocity Meter 9545 (TSI, Dallas, TX, USA) thermo-anemometer was used to establish the temperature, relative humidity, and airflow rate at the selected workstations. The measurements were taken over 2 min at 1 s intervals; averages were logged for each sampling variant (day/hour/location).

The concentration of particulate matter (PM_1_; PM_2.5_; PM_4_; PM_10_; PM_total_) was measured using a DustTrak™ DRX Aerosol Monitor 8533 portable laser photometer (TSI, USA). The detection range for particles with diameters ranging from 0.1 to 15 μm was between 0.001 and 150 mg m^−3^. The measurements were carried out in triplicate for each location at 1.5 m from the ground level. The sampling rate was set to 3 L min^−1^ and the sampling interval to 5 s. The total sampling time was 3 min. The carbon dioxide and formaldehyde concentrations were measured using a M200 Multi-functional Air Quality Detector (Temtop, China).

### 3.3. Volatile Compounds Analysis

Detailed analysis of the volatile compounds was carried out using headspace solid-phase microextraction coupled to gas chromatography-mass spectrometry (HS-SPME-GC). Tedlar bags (5 L) were used for the collection of air samples from Rooms no. 2 and 6 (gym and external background). For extraction of the volatile compounds from the air samples, the solid-phase microextraction technique was used with the fiber covered with 50/30 μm divinylbenzene/carboxen/polydimethylsiloxane (DVB/CAR/PDMS) phase (length 1 cm). The SPME fiber was inserted via the sampling port, followed by exposure for 60 min at 20 °C. After the adsorption of volatiles, the fiber was retracted into the needle and transferred to the inlet of the GC apparatus for the desorption of analytes. Desorption was carried out for 5 min at 250 °C. Before each extraction, the fiber was heated for 10 min in the inlet of the GC apparatus at 260 °C for cleaning. A GC-MS system was used for the volatile compound analysis (GC Agilent 7890A and MS Agilent MSD 5975C, Agilent Technologies, Santa Clara, CA, USA). The compounds were separated on a capillary column DB-1ms 60 m × 0.25 mm × 0.25 µm (Agilent Technologies, Santa Clara, CA, USA). All injections were performed in a splitless mode. As a carrier gas, helium was used with a flow rate of 1.1 mL/min. The GC oven temperature was programmed to increase from 30 °C (10 min) to 70 °C at a rate of 2 °C/min and kept for 2 min, then to 235 °C at a rate of 10 °C/min, and finally kept for 3.5 min. The MS ion source, transfer line, and quadrupole analyzer temperatures were 230, 250, and 150 °C, respectively. The electron impact energy was set at 70 eV. The mass spectrometer was operated in full scan mode (SCAN). The qualification of volatiles was performed by a comparison of the obtained spectra with the reference mass spectra from the NIST/EPA/NIH mass spectra library (2012; Version 2.0 g) or with mass spectra obtained from the GC standards and confirmed with the use of the deconvolution procedure. Then, retention indices (RI) were calculated according to the formula proposed by van den Dool and Kratz [85] relative to a homologous series of n-alkanes from C5 to C20. Retention indices were compared with the literature data [86]. Data processing was conducted with Mass Hunter Workstation Software (Agilent, Santa Clara, CA, USA). The relative amounts of volatile compounds were calculated by the individual peak area relative to the total peak areas.

### 3.4. Determination of Airborne Microorganism Number

Air samples were collected in triplicate (20–100 L) per sampling site at the height of about 1.5 m from ground level with an airflow rate of 100 L min^−1^ using a MAS-100 Eco Air Sampler (Merck, Darmstadt, Germany), according to EN 13098 [87]. The microbiological contamination of the air was determined using: TSA (tryptic soy agar, Merck, Germany) with (0.2%) nystatin to determine the number of bacteria and MEA (MALT EXTRACT agar, Merck, Germany) medium with (0.1%) chloramphenicol to determine the fungi number. The samples were incubated at either 25 ± 2 °C for 5–7 days (fungi) or 30 ± 2 °C for 48 h (bacteria). After incubation, the colonies were counted and corrected based on Feller’s statistical correction table. The results were calculated as the arithmetic mean of three independent repetitions and expressed in CFU m^−3^.

### 3.5. Determination of Surface Microbial Contamination

Samples from 20 different surfaces throughout the facility (two independent repetitions) were collected on the first day of testing (Monday) between 8:00 and 10:00 using Hygicult^®^ TPC (Orion Diagnostica Oy, Espoo, Finland) with the Total Plate Count medium. The collected samples were incubated at 30 ± 2 °C for 3–5 days. Next, the colonies were counted, and the results (arithmetic mean of two independent repetitions) were expressed in CFU cm^−2^.

### 3.6. Detection of SARS-CoV-2

Swabs were taken from approx. 100 cm^2^ from three surfaces (treadmill touch panel, panel and grips of elliptical cross trainer, and multi-gym grips) located in the gym (Room no. 2) using R9F buffer (A&A Biotechnology, Gdańsk, Poland). The surfaces were selected based on the highest frequency of use, the presence of direct contact with the user’s hands, and their vicinity to the breathing zone. RNA isolation was performed with the CoV RNA Kit (A&A Biotechnology, Poland). The presence of the SARS-CoV-2 virus RNA in the tested samples was confirmed by Real-Time PCR with Taq-Man probes. The presence of SARS-CoV-2 was tested using the MediPAN-2G+ FAST COVID test (Medicofarma, Warsaw, Poland) kit by A&A Biotechnology (Poland) according to the manufacturer’s instructions. The test detects fragments of two SARS-CoV-2 genes (i.e., ORF1ab (nsp2) and gene S). A synthetic fragment of a plant virus genome was used as a control.

### 3.7. Determination of Biodiversity

Dust deposited on the surface of the gym equipment (10–12 devices located 0.5–2 m from the ground) was collected with steely, dry swabs and refrigerated overnight (4 °C). Then, the samples were combined into one and used for DNA extraction. According to the manufacturer’s instructions, genomic DNA was extracted using the Soil DNA Purification Kit (EURX, Poland). The presence of genomic DNA in the tested samples was confirmed with fluorimetry (Qubit). The extracted DNA concentration was 1 µg mL^−1^. Universal primers amplifying the 16S rRNA bacterial gene’s fragment and fungal ITS regions were used in the reaction [88,89,90]. Q5 Hot Start High-Fidelity 2X Master Mix (NEB, Ipswich, MA, USA) was used for PCR according to the manufacturer’s instructions. The libraries were prepared and sequenced by Genomed (Warsaw, Poland) using the paired-end technology on the Illumina MiSeq (2 × 300 nt) platform with the use of a v3 Kit (Illumina, San Diego, CA, USA). Automatic initial analysis was performed on the MiSeq sequencer using MiSeq Reporter (MSR) v2.6. The obtained results were next subjected to bioinformatic analysis. Adapter sequences were removed from the reads, which were next subjected to quality control with the Cutadapt program using quality (<20) and the minimal length of (30 nt) threshold [91]. Library reads 16S were further processed using the DADA2 package to separate sequences of biological origin from those generated during the sequencing process. This package was also used for selecting unique sequences of biological origin, the so-called amplicon sequence variant (ASV). Bioinformatics analysis of the reads for species-level classification was performed using the QIIME 2 program based on the Silva 138 database using a hybrid approach [92]. First, ASV sequences were compared with the database to find identical reference sequences using the VSEARCH algorithm [93]. Next, the atypical sequences left over from the previous step were classified based on machine learning, which was performed using SKLearn. ITS library reads classification at the species level was performed using QIIME based on the UNITE v8 reference database [94]. After filtering, as described above, the reads were clustered based on the reference database using the UCLUST algorithm. Chimeric sequences were removed using the USEARCH (usearch61) algorithm. Finally, the taxonomy was assigned to the reference database using the BLAST algorithm. Sequencing data files in the FASTQ format were deposited in the NCBI Sequence Read Archive (SRA) under BioProject accession number PRJNA818521 (BioSampleAcc. SAMN26866224 and Run Acc. SRR18428312 and SRR18428311).

### 3.8. Statistical Analysis

Statistical analysis was carried out with Statistica 13.1 (Statsoft, Tulsa, OK, USA). Descriptive statistics were calculated for all variables of interest. For the microclimate parameters and the number of microorganisms in the air, one-way analysis of variance (ANOVA) was performed for data grouped depending on the sampling day, hour, and location. ANOVA assumptions were checked with the Shapiro–Wilk and Levene tests. When a statistical difference was detected, the means were compared using Tukey’s post hoc test or Dunn’s post hoc tests. Full-factorial ANOVA was performed for the particulate matter concentration, followed by Tukey’s post hoc test. In the case of surface microbial contamination, the Fisher–Snedecor test was carried out for the number of microorganisms averaged over the tested surfaces. The variances in the number of bacteria and fungi on the examined surfaces were heterogeneous. Thus, a *t*-test was performed for unequal variances. All tests were performed at a significance level of 0.05.

Linear regression was performed to check a correlation between the number of bacteria and fungi in the air and other measured parameters. To describe the strength of the correlation, the Evans (1996) guide for the absolute value of correlation coefficient r: 0.00–0.19 “very weak”; 0.20–0.39 “weak”; 0.40–0.59 “moderate”; 0.60–0.79 “strong”; 0.80–1.0 “very strong” [95] was used.

## 4. Conclusions

High particulate matter, especially the PM_2.5_ concentration, was observed in fitness centers that exceeded the environmental threshold and supports statements that air quality inside sports facilities can be worse than that outdoors. Moreover, chemical markers such as CO_2_ concentration and VOCs (phenol, toluene, and 2-ethyl-1-hexanol) may be useful for air quality monitoring in sports facilities.

Additionally, the concentration of airborne microorganisms was high compared to the previous research and literature recommendations. It is also noteworthy that genera of bacteria (*Escherichia-Shigella*, *Corynebacterium*, *Bacillus*, *Staphylococcus*) and fungi (*Cladosporium*, *Aspergillus*, *Penicillium*), potentially belonging to the second and third groups of health hazards following Directive 2019/1833/EC, were detected, albeit at relatively low concentrations. In addition, other species that may be allergenic (*Epicoccum*) or infectious (*Acinetobacter*, *Sphingomonas*, *Sporobolomyces*) and the SARS-CoV-2 virus were detected.

Due to the possibility of high contamination with chemicals, CO_2_, bacteria and fungi, and the spread of the SARS-CoV-2 virus in sports facilities, air purification systems with proven effectiveness are also needed (e.g., UV flow lamps, photocatalytic ionizers) for continuous operation during opening hours. Future research should aim at introducing systems of constant air quality monitoring in sports facilities (e.g., using multiple sensors including microfluidic chips) and developing warning systems against exceeding the concentration of suspended dust or the recommended number of microorganisms in the air.

## Figures and Tables

**Table 1:** Air quality parameters at the tested locations.

Room No.	Description	Parameter					
		Temperature, °C	Relative Humidity, %	Air Velocity m/s	Total PM Concentration, mg/m^3^	CO_2_ Concentration, ppm	HCN Concentration, mg/m^3^
1	Reception	M: 25.63 ^a^	M: 58.52 ^ab^	M: 0.092 ^bdef^	M: 0.058 ^a^	M: 800 ^ab^	M: 0.005 ^a^
		SD: 3.57	SD: 10.23	SD: 0.153	SD: 0.009	SD: 94	SD: 0.006
2	The gym	M: 25.28 ^ab^	M: 53.73 ^b^	M: 0.072 ^acf^	M: 0.057 ^a^	M: 2198 ^c^	M: 0.049 ^c^
		SD: 0.95	SD: 6.99	SD: 0.057	SD: 0.010	SD: 111	SD: 0.002
3	Fitness room on the first floor	M: 24.82 ^b^	M: 57.62 ^ab^	M: 0.067 ^aceg^	M: 0.057 ^a^	M: 1773 ^ad^	M: 0.042 ^ab^
		SD: 0.81	SD: 8.16	SD: 0.053	SD: 0.009	SD: 51	SD: 0.001
4	Fitness room on the second floor	M: 25.49 ^ab^	M: 62.31 ^a^	M: 0.025 ^bdg^	M: 0.059 ^a^	M: 2017 ^cd^	M: 0.047 ^bc^
		SD: 0.75	SD: 6.07	SD: 0.024	SD: 0.010	SD: 32	SD: 0.001
5	Women’s cloakroom	M: 26.18 ^a^	M: 60.82 ^ab^	M: 0.019 ^b^	M: 0.057 ^a^	M: 1925 ^bcd^	M: 0.046 ^abc^
		SD: 0.67	SD: 5.84	SD: 0.008	SD: 0.010	SD: 67	SD: 0.001
6	Atmospheric air (external background)	M: 26.40 *^ab^	M: 70.00 *^a^	M: 5.125 *^a^	M: 0.024 **^b^	M: 593 ^a^	M: 0.067 ^c^
		SD: 2.30	SD: 5.24	SD: 0.978	SD: 0.004	SD: 44	SD: 0.027

M—mean; SD—standard deviation; statistically different samples were marked with different letters within the same column (Kruskal–Wallis test followed by Dunn’s post hoc tests at a significance level of 0.05); data sources for the atmospheric air: (*) https://www.ekologia.pl/pogoda/polska/lodzkie/zdunska-wola/archiwum,zakres (accessed on 26 July 2012), (**) https://powietrze.gios.gov.pl/pjp/current/station_details/archive/350# (accessed on 26 July 2012).

**Table 2:** Detection of SARS-CoV-2 on the fitness center surfaces.

Sample No.	Description	RNA Concentration, μg/mL	SARS-CoV-2 RNA
1	Treadmill touch panel	66	Present
2	Panel and grips of elliptical cross trainer	63	Absent
3	Multi-gym grips panel	63	Absent

**Table 3:** Characteristics of the sampling sites at points in the tested fitness center.

Room No.	Description	Area, m^2^	Surface Sampling Sites	Number of Users	Number of Ceiling Fans/Opening Windows	Airconditioning	Number of Samples
1	Reception	18	Table for the waiting, receptionist’s desk, armrest on the couch, chair	2–12	1/1	No	N = 60 n = 4
2	The gym	220	Treadmill touch panel, the touch panel of the cross trainer, mirror, thick mat on the floor	1–9	3/6	Yes (2 units)	N = 60 n = 4
3	Fitness room on the first floor	100	Thin exercise mat, MMA bag, small “ovoball”, 1 kg dumbbell holder	1–11	2/5	Yes (1 unit)	N = 60 n = 4
4	Fitness room on the second floor	85	Large exercise ball, exercise bike saddle, trampoline handle, 8 kg dumbbell holder	1–12	2/6	Yes (1 unit)	N = 60 n = 4
5	Women’s cloakroom	26	Mirror, the bottom of the locker, the external part of the locker, shoe cabinet	1–5	1/0	No	N = 60 n = 4
6	Atmospheric air (external background)	-	Parking lot located 10 m from the entrance to the fitness club building	-	-	-	N = 60 n = 0

“-”—not applicable; N—number of air samples collected during the whole working week, collected in triplicate; n—total number of surface samples.

## Data Availability

The data that support the findings of this study are available on request from the corresponding author.

## Supplementary Materials

The following supporting information can be downloaded at: https://www.mdpi.com/article/10.3390/molecules28083560/s1, Table S1: Number–size distribution of airborne particles detected at the tested locations. Table S2. Volatile compounds identified in the gym; Table S3: The average number of microorganisms in the tested fitness club; Figure S1. Microclimatic conditions (means with SD): (a) daily averages, (b) averaged over sampling time of the day, (c) averaged over sampling location; statistically different samples were marked with different letters; no letters indicate no statistical differences (Tukey’s test, α = 0.05); Figure S2. Averaged values of the total PM concentration (means with SD): (a) daily, (b) over sampling time of the day, (c) over sampling location; statistically different samples were marked with different letters; (Tukey’s test, α = 0.05); Figure S3: Phylogenetic distribution of hazardous bacteria sequences assigned to genera in the settled dust sample; Figure S4: Phylogenetic distribution of hazardous fungi sequences assigned to genera in the settled dust sample.

## Sample Availability

Samples are available from the authors.

## References

[1] Małecka-Adamowicz M., Kubera Ł., Jankowiak E., Dembowska E. (2019). Microbial diversity of bioaerosol inside sports facilities and antibiotic resistance of isolated *Staphylococcus* spp.. Aerobiologia.

[2] Ramos C.A., Wolterbeek H.T., Almeida S.M. (2014). Exposure to indoor air pollutants during physical activity in fitness centers. Build. Environ..

[3] WHO Physical Activity Fact Sheet. https://www.who.int/publications/i/item/WHO-HEP-HPR-RUN-2021.2.

[4] Deloitte Sports Retail Study 2020. Findings from a Central European Consumer Survey. https://www2.deloitte.com/pl/pl/pages/consumer-business/articles/sports-retail-study-2020.html.

[5] Boonrattanakij N., Yomchinda S., Lin F.-J., Bellotindos L.M., Lu M.-C. (2021). Investigation and disinfection of bacteria and fungi in sports fitness center. Environ. Sci. Pollut. Res..

[6] Saini J., Dutta M., Marques G. (2020). A comprehensive review on indoor air quality monitoring systems for enhanced public health. Sustain. Environ. Res..

[7] Szulc J., Cichowicz R., Gutarowski M., Okrasa M., Gutarowska B. (2023). Assessment of Dust, Chemical, Microbiological Pollutions and Microclimatic Parameters of Indoor Air in Sports Facilities. Int. J. Environ. Res. Public Health.

[8] Bralewska K., Rogula-Kozłowska W., Bralewski A. (2022). Indoor air quality in sports center: Assessment of gaseous pollutants. Build. Environ..

[9] Finewax Z., Pagonis D., Claflin M.S., Handschy A.V., Brown W.L., Jenks O., Nault B.A., Day D.A., Lerner B.M., Jimenez J.L. (2021). Quantification and source characterization of volatile organic compounds from exercising and application of chlorine-based cleaning products in a university athletic center. Indoor Air.

[10] Mbareche H., Morawska L., Duchaine C. (2019). On the interpretation of bioaerosol exposure measurements and impacts on health. J. Air Waste Manag. Assoc..

[11] Kim K.-H., Kabir E., Jahan S.A. (2018). Airborne bioaerosols and their impact on human health. J. Environ. Sci..

[12] Maus R., Goppelsröder A., Umhauer H. (2001). Survival of bacterial and mold spores in air filter media. Atmos. Environ..

[13] Kalogerakis N., Paschali D., Lekaditis V., Pantidou A., Eleftheriadis K., Lazaridis M. (2005). Indoor air quality—Bioaerosol measurements in domestic and office premises. J. Aerosol Sci..

[14] Prussin A.J., Marr L.C. (2015). Sources of airborne microorganisms in the built environment. Microbiome.

[15] Haghverdian B.A., Patel N., Wang L., Cotter J.A. (2018). The sports ball as a fomite for transmission of Staphylococcus aureus. J. Environ. Health.

[16] CDC Centers for Disease Control and Prevention Methicillin-Resistant Staphylococcus Aureus (MRSA). https://www.cdc.gov/mrsa/index.html.

[17] Bragoszewska E., Biedroń I., Mainka A. (2020). Microbiological air quality in a highschool gym located in an urban area of Southern Poland-preliminary research. Atmosphere.

[18] Kic P., Ruzek L., Popelářová E. (2018). Concentration of air-borne microorganisms in sport facilities. Agron. Res..

[19] Fadare O.S., Durojaye O.B. (2019). Antibiotic Susceptibility Profile of Bacteria Isolated from Fitness Machines in Selected Fitness Centers at Akure and Elizade University in Ondo State Nigeria. Microbiol. Res. J. Int..

[20] Mukherjee N., Dowd S., Wise A., Kedia S., Vohra V., Banerjee P. (2014). Diversity of Bacterial Communities of Fitness Center Surfaces in a U.S. Metropolitan Area. Int. J. Environ. Res. Public Health.

[21] Huang C., Que J., Liu Q., Zhang Y. (2021). On the gym air temperature supporting exercise and comfort. Build. Environ..

[22] International Fitness Association Gym Temperature and Noise Standards. https://www.ifafitness.com/health/temperature.htm.

[23] Zhai Y., Elsworth C., Arens E., Zhang H., Zhang Y., Zhao L. (2015). Using air movement for comfort during moderate exercise. Build. Environ..

[24] (2019). Bralewska; Rogula-Kozłowska; Bralewski Size-Segregated Particulate Matter in a Selected Sports Facility in Poland. Sustainability.

[25] (2008). Directive 2008/50/EC of the European Parliament and of the Council of 21 May 2008 on ambient air quality and cleaner air for Europe. Off. J. Eur. Union.

[26] Andrade A., Dominski F.H., Coimbra D.R. (2017). Scientific production on indoor air quality of environments used for physical exercise and sports practice: Bibliometric analysis. J. Environ. Manag..

[27] Rundell K.W., Caviston R. (2008). Ultrafine and Fine Particulate Matter Inhalation Decreases Exercise Performance in Healthy Subjects. J. Strength Cond. Res..

[28] Cutrufello P.T., Smoliga J.M., Rundell K.W. (2012). Small Things Make a Big Difference. Sport. Med..

[29] Pope C.A., Burnett R.T., Thurston G.D., Thun M.J., Calle E.E., Krewski D., Godleski J.J. (2004). Cardiovascular Mortality and Long-Term Exposure to Particulate Air Pollution. Circulation.

[30] Daigle C.C., Chalupa D.C., Gibb F.R., Morrow P.E., Oberdörster G., Utell M.J., Frampton M.W. (2003). Ultrafine Particle Deposition in Humans During Rest and Exercise. Inhal. Toxicol..

[31] Castro A., Calvo A.I., Alves C., Alonso-Blanco E., Coz E., Marques L., Nunes T., Fernández-Guisuraga J.M., Fraile R. (2015). Indoor aerosol size distributions in a gymnasium. Sci. Total Environ..

[32] Phalen R.F. (2009). Inhalation Studies: Foundations and Techniques.

[33] World Health Organization (2010). WHO Guidelines for Air Quality: Selected Pollutants.

[34] Environmental Protection Agency National Ambient Air Quality Standards (NAAQS). https://www.epa.gov/sites/default/files/2015-02/documents/criteria.pdf.

[35] Issitt T., Wiggins L., Veysey M., Sweeney S.T., Brackenbury W.J., Redeker K. (2022). Volatile compounds in human breath: Critical review and meta-analysis. J. Breath Res..

[36] Jahn L.G., Tang M., Blomdahl D., Bhattacharyya N., Abue P., Novoselac A., Ruiz L.H., Misztal P.K. (2023). Volatile organic compound (VOC) emissions from the usage of benzalkonium chloride and other disinfectants based on quaternary ammonium compounds. Environ. Sci. Atmos..

[37] Singal M., Vitale D., Smith L. (2011). Fragranced Products and VOCs. Environ. Health Perspect..

[38] Sherzad M., Jung C. (2022). Evaluating the emission of VOCs and HCHO from furniture based on the surface finish methods and retention periods. Front. Built Environ..

[39] Phenol and Phenolic Compound. https://cpcb.nic.in/uploads/News_Letter_Phenols_Phenolic_Compounds_2017.pdf.

[40] European Chemicals Agency Phenol. https://echa.europa.eu/pl/substance-information/-/substanceinfo/100.003.303.

[41] Michałowicz J., Duda W. (2007). Phenols—Sources and toxicity. Polish J. Environ. Stud..

[42] Pytel K., Marcinkowska R., Zabiegała B. (2021). Investigation on air quality of specific indoor environments—Spa salons located in Gdynia, Poland. Environ. Sci. Pollut. Res..

[43] Gonçalves A.D., Martins T.G., Cassella R.J. (2021). Passive sampling of toluene (and benzene) in indoor air using a semipermeable membrane device. Ecotoxicol. Environ. Saf..

[44] Wakayama T., Ito Y., Sakai K., Miyake M., Shibata E., Ohno H., Kamijima M. (2019). Comprehensive review of 2-ethyl-1-hexanol as an indoor air pollutant. J. Occup. Health.

[45] European Commission—Employment Social Affairs & Inclusion Recommendation from the Scientific Committee on Occupational Exposure Limits for 2-Ethylhexanol. https://www.google.com/url?sa=t&rct=j&q=&esrc=s&source=web&cd=&cad=rja&uact=8&ved=2ahUKEwiX7PGYnLP-AhXis4sKHREOAH8QFnoECA0QAQ&url=https%3A%2F%2Fec.europa.eu%2Fsocial%2FBlobServlet%3FdocId%3D6660%26langId%3Den&usg=AOvVaw0sUX_qCunAQmBjIZhWuHFe.

[46] Skowroń J., Górny R.L., Pośniak M., Skowroń J. (2020). Harmful biological agents. The Interdepartmental Commission for Maximum Admissible Concentrations and Intensities for Agents Harmful to Health in the Working Environment: Limit Values 2020.

[47] World Health Organization (2006). Air Quality Guidelines for Particulate Matter, Ozone, Nitrogen Dioxide and Sulfur Dioxide: Global Update 2005: Summary of Risk Assessment.

[48] Adams R.I., Bhangar S., Pasut W., Arens E.A., Taylor J.W., Lindow S.E., Nazaroff W.W., Bruns T.D. (2015). Chamber bioaerosol study: Outdoor air and human occupants as sources of indoor airborne microbes. PLoS ONE.

[49] Frankel M., Bekö G., Timm M., Gustavsen S., Hansen E.W., Madsen A.M. (2012). Seasonal Variations of Indoor Microbial Exposures and Their Relation to Temperature, Relative Humidity, and Air Exchange Rate. Appl. Environ. Microbiol..

[50] Skóra J., Gutarowska B., Pielech-Przybylska K., Stępień Ł., Pietrzak K., Piotrowska M., Pietrowski P. (2015). Assessment of microbiological contamination in the work environments of museums, archives and libraries. Aerobiologia.

[51] European Commission (2019). Commission Directive (EU) 2019/1833 of 24 October 2019 Amending Annexes I, III, V and VI to Directive 2000/54/EC of the European Parliament and of the Council as Regards Purely Technical Adjustments.

[52] Żyrek D. (2019). Ocena skażenia mikrobiologicznego powierzchni sprzętu do ćwiczeń w siłowniach. Forum Zakażeń.

[53] Turkstani M.A., Sultan R.M.S., Al-Hindi R.R., Ahmed M.M.M. (2021). Molecular identification of microbial contaminations in the fitness center in Makkah region. Biosci. J..

[54] Boa T.T., Rahube T.O., Fremaux B., Levett P.N., Yost C.K. (2013). Prevalence of methicillin-resistant staphylococci species isolated from computer keyboards located in secondary and postsecondary schools. J. Environ. Health.

[55] Lasek R., Szuplewska M., Mitura M., Decewicz P., Chmielowska C., Pawłot A., Sentkowska D., Czarnecki J., Bartosik D. (2018). Genome Structure of the Opportunistic Pathogen *Paracoccus yeei* (Alphaproteobacteria) and Identification of Putative Virulence Factors. Front. Microbiol..

[56] Daneshvar M.I., Hollis D.G., Weyant R.S., Steigerwalt A.G., Whitney A.M., Douglas M.P., Macgregor J.P., Jordan J.G., Mayer L.W., Rassouli S.M. (2003). *Paracoccus yeeii* sp. nov. (Formerly CDC Group EO-2), a Novel Bacterial Species Associated with Human Infection. J. Clin. Microbiol..

[57] Cao Y.-R., Jiang Y., Wang Q., Tang S.-K., He W.-X., Xue Q.-H., Xu L.-H., Jiang C.-L. (2010). *Rubellimicrobium roseum* sp. nov., a Gram-negative bacterium isolated from the forest soil sample. Antonie Van Leeuwenhoek.

[58] Leys N.M.E.J., Ryngaert A., Bastiaens L., Verstraete W., Top E.M., Springael D. (2004). Occurrence and Phylogenetic Diversity of Sphingomonas Strains in Soils Contaminated with Polycyclic Aromatic Hydrocarbons. Appl. Environ. Microbiol..

[59] El Beaino M., Fares J., Malek A., Hachem R. (2018). Sphingomonas paucimobilis-related bone and soft-tissue infections: A systematic review. Int. J. Infect. Dis..

[60] Moura J.B., Delforno T.P., do Prado P.F., Duarte I.C. (2021). Extremophilic taxa predominate in a microbial community of photovoltaic panels in a tropical region. FEMS Microbiol. Lett..

[61] Premalatha N., Gopal N.O., Jose P.A., Anandham R., Kwon S.W. (2015). Optimization of cellulase production by *Enhydrobacter* sp. ACCA2 and its application in biomass saccharification. Front. Microbiol..

[62] Viegas C., Alves C., Carolino E., Rosado L., Silva Santos C. (2010). Prevalence of Fungi in Indoor Air with Reference to Gymnasiums with Swimming Pools. Indoor Built Environ..

[63] Zhu L., Li T., Xu X., Shi X., Wang B. (2021). Succession of Fungal Communities at Different Developmental Stages of Cabernet Sauvignon Grapes from an Organic Vineyard in Xinjiang. Front. Microbiol..

[64] Tsuji M., Tanabe Y., Vincent W.F., Uchida M. (2019). *Vishniacozyma ellesmerensis* sp. nov., a psychrophilic yeast isolated from a retreating glacier in the Canadian High Arctic. Int. J. Syst. Evol. Microbiol..

[65] Riebesehl J., Yurchenko E., Nakasone K.K., Langer E. (2019). Phylogenetic and morphological studies in Xylodon (Hymenochaetales, Basidiomycota) with the addition of four new species. MycoKeys.

[66] Markson A.A., Akwaji P.I., Umana E.J. (2017). Mushroom Biodiversity of Cross River National Park (Oban Hills Division), Nigeria. World Sci. News.

[67] Aronsen A., Læssøe T. (2016). Fungi of Northern Europe, Volume 5: The Genus Mycena s.l..

[68] de Hoog G.S., Guarro J., Gené J., Ahmed S., Al-Hatmi A.M.S., Figueras M.J., Vitale R.G. (2001). Atlas of Clinical Fungi.

[69] Damji R., Mukherji A., Mussani F. (2019). Sporobolomyces salmonicolor: A case report of a rare cutaneous fungal infection. SAGE Open Med. Case Rep..

[70] Ilinsky Y., Lapshina V., Verzhutsky D., Fedorova Y., Medvedev S. (2022). Genetic Evidence of an Isolation Barrier between Flea Subspecies of *Citellophilus tesquorum* (Wagner, 1898) (Siphonaptera: Ceratophyllidae). Insects.

[71] Rybitwa D., Wawrzyk A., Rahnama M. (2020). Application of a Medical Diode Laser (810 nm) for Disinfecting Small Microbiologically Contaminated Spots on Degraded Collagenous Materials for Improved Biosafety in Objects of Exceptional Historical Value From the Auschwitz-Birkenau State Museum and Prot. Front. Microbiol..

[72] Rybitwa D., Wawrzyk A., Wilczyński S., Łobacz M. (2020). Irradiation with medical diode laser as a new method of spot-elimination of microorganisms to preserve historical cellulosic objects and human health. Int. Biodeterior. Biodegrad..

[73] Gutarowska B., Szulc J., Nowak A., Otlewska A., Okrasa M. (2018). Dust at various workplaces-microbiological and toxicological threats. Int. J. Environ. Res. Public Health.

[74] Edet U., Antai S., Brooks A., Asitok A., Enya O., Japhet F. (2017). An Overview of Cultural, Molecular and Metagenomic Techniques in Description of Microbial Diversity. J. Adv. Microbiol..

[75] Polish Ministry of Health Coronavirus Infections Report (SARS-CoV-2). https://www.gov.pl/web/koronawirus/wykaz-zarazen-koronawirusem-sars-cov-2.

[76] Chu D.K.W., Gu H., Chang L.D.J., Cheuk S.S.Y., Gurung S., Krishnan P., Ng D.Y.M., Liu G.Y.Z., Wan C.K.C., Tsang D.N.C. (2021). SARS-CoV-2 Superspread in Fitness Center, Hong Kong, China, March 2021. Emerg. Infect. Dis..

[77] Li H., Shankar S.N., Witanachchi C.T., Lednicky J.A., Loeb J.C., Alam M.M., Fan Z.H., Mohamed K., Eiguren-Fernandez A., Wu C.-Y. (2021). Environmental Surveillance and Transmission Risk Assessments for SARS-CoV-2 in a Fitness Center. Aerosol Air Qual. Res..

[78] Lendacki F.R., Teran R.A., Gretsch S., Fricchione M.J., Kerins J.L. (2021). COVID-19 Outbreak Among Attendees of an Exercise Facility—Chicago, Illinois, August–September 2020. MMWR. Morb. Mortal. Wkly. Rep..

[79] Helsingen L.M., Løberg M., Refsum E., Gjøstein D.K., Wieszczy P., Olsvik Ø., Juul F.E., Barua I., Jodal H.C., Herfindal M. (2021). COVID-19 transmission in fitness centers in Norway—A randomized trial. BMC Public Health.

[80] Salonen H., Salthammer T., Morawska L. (2020). Human exposure to air contaminants in sports environments. Indoor Air.

[81] Masotti F., Cattaneo S., Stuknytė M., De Noni I. (2019). Airborne contamination in the food industry: An update on monitoring and disinfection techniques of air. Trends Food Sci. Technol..

[82] Vasilyak L.M. (2021). Physical Methods of Disinfection (A Review). Plasma Phys. Rep..

[83] Song X., Vossebein L., Zille A. (2019). Efficacy of disinfectant-impregnated wipes used for surface disinfection in hospitals: A review. Antimicrob. Resist. Infect. Control.

[84] Viana Martins C.P., Xavier C.S.F., Cobrado L. (2022). Disinfection methods against SARS-CoV-2: A systematic review. J. Hosp. Infect..

[85] van Den Dool H., Kratz P.D. (1963). A generalization of the retention index system including linear temperature programmed gas—Liquid partition chromatography. J. Chromatogr. A.

[86] NIST Chemistry WebBook. https://webbook.nist.gov/chemistry/.

[87] (2019). Workplace Exposure—Measurement of Airborne Microorganisms and Microbial Compounds—General Requirements.

[88] Schmidt P.-A., Bálint M., Greshake B., Bandow C., Römbke J., Schmitt I. (2013). Illumina metabarcoding of a soil fungal community. Soil Biol. Biochem..

[89] Vilgalys R., Gonzalez D. (1990). Organization of ribosomal DNA in the basidiomycete Thanatephorus praticola. Curr. Genet..

[90] Klindworth A., Pruesse E., Schweer T., Peplies J., Quast C., Horn M., Glöckner F.O. (2013). Evaluation of general 16S ribosomal RNA gene PCR primers for classical and next-generation sequencing-based diversity studies. Nucleic Acids Res..

[91] Martin M. (2011). Cutadapt removes adapter sequences from high-throughput sequencing reads. EMBnet J..

[92] Bolyen E., Rideout J.R., Dillon M.R., Bokulich N.A., Abnet C.C., Al-Ghalith G.A., Alexander H., Alm E.J., Arumugam M., Asnicar F. (2019). Reproducible, interactive, scalable and extensible microbiome data science using QIIME 2. Nat. Biotechnol..

[93] Rognes T., Flouri T., Nichols B., Quince C., Mahé F. (2016). VSEARCH: A versatile open source tool for metagenomics. PeerJ.

[94] Kõljalg U., Nilsson H.R., Schigel D., Tedersoo L., Larsson K.-H., May T.W., Taylor A.F.S., Jeppesen T.S., Frøslev T.G., Lindahl B.D. (2020). The Taxon Hypothesis Paradigm—On the Unambiguous Detection and Communication of Taxa. Microorganisms.

[95] Evans J.D. (1995). Straightforward Statistics for the Behavioral Sciences.

