# Supplementary material for: Markers of Chemical and Microbiological Contamination of the Air in the Sport Centers
Source: Molecules. 2023 Apr 18;28(8):3560. doi: 10.3390/molecules28083560 (PMC10144153; doi:10.3390/molecules28083560)
Supplement: Supplementary file 1 [file molecules-28-03560-s001.zip › Figure S1.pdf]

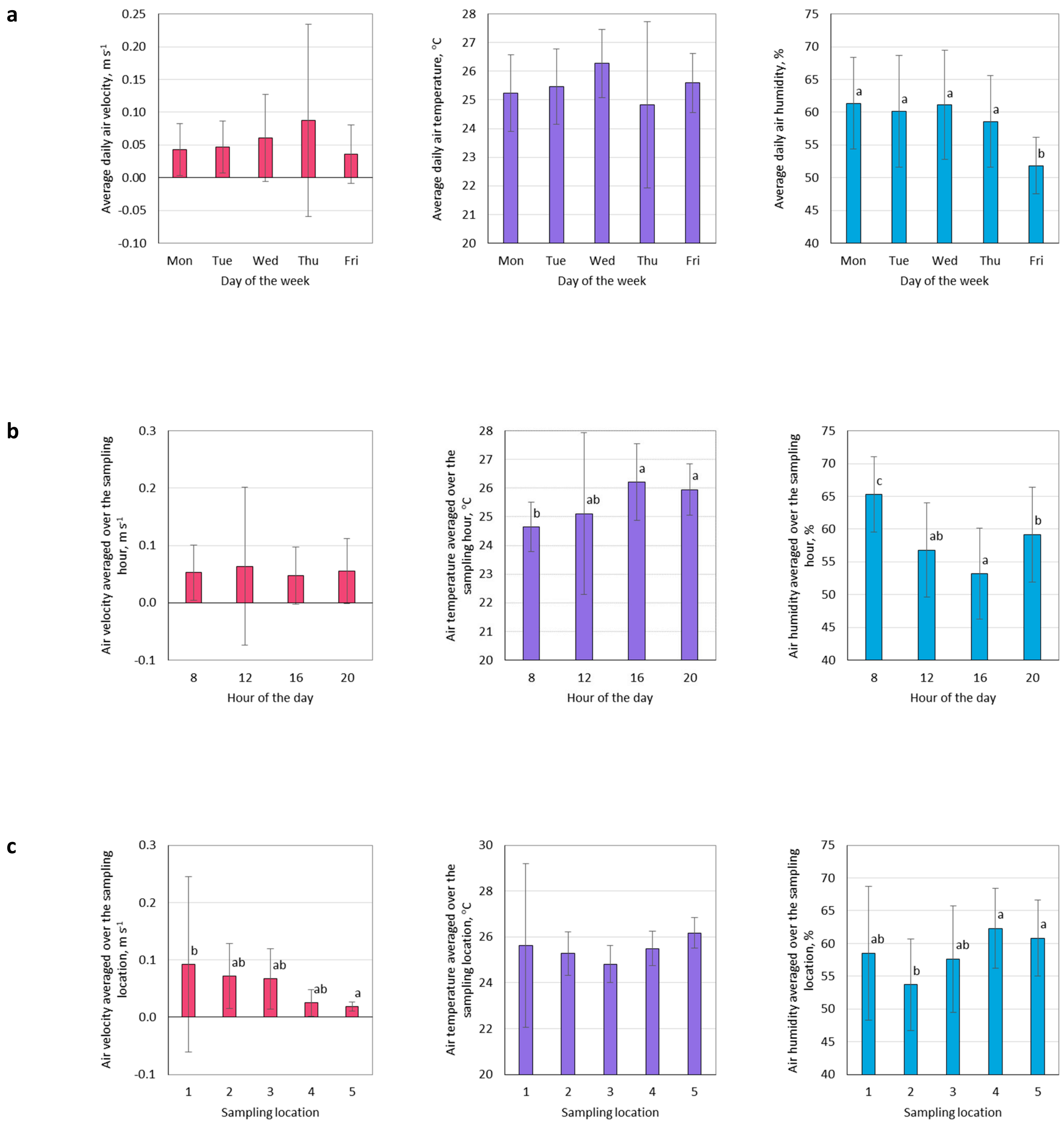

Figure S1. Microclimatic conditions (means with SD): a) daily averages, b) averaged over sampling time of the day, c) averaged over sampling location; statistically different samples were marked with different letters; no letters indicate no statistical differences (Tukey's test, α=0.05).
