# Supplementary material for: Markers of Chemical and Microbiological Contamination of the Air in the Sport Centers
Source: Molecules. 2023 Apr 18;28(8):3560. doi: 10.3390/molecules28083560 (PMC10144153; doi:10.3390/molecules28083560)
Supplement: Supplementary file 1 [file molecules-28-03560-s001.zip › Figure S2.pdf]

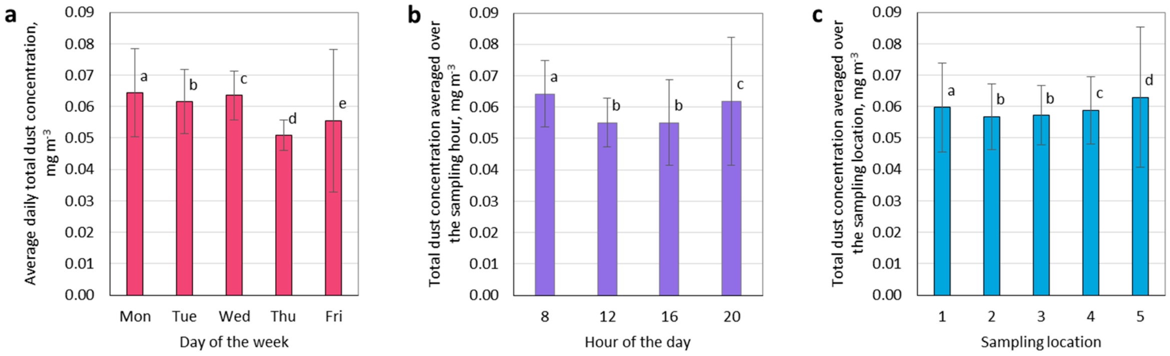

Figure S2. Averaged values of total PM concentration (means with SD): a) daily, b) over sampling time of the day, c) over sampling location; statistically different samples were marked with different letters; (Tukey's test, α=0.05).
