# Supplementary material for: Markers of Chemical and Microbiological Contamination of the Air in the Sport Centers
Source: Molecules. 2023 Apr 18;28(8):3560. doi: 10.3390/molecules28083560 (PMC10144153; doi:10.3390/molecules28083560)
Supplement: Supplementary file 1 [file molecules-28-03560-s001.zip › Table S1.pdf]

**Table S1.** Number-size distribution of airborne particles detected at tested locations.

| Day of the | Hour | Locatio | Number of particles per bin* | | | | | | | | | | | | | | | | |
|---|---|---|---|---|---|---|---|---|---|---|---|---|---|---|---|---|---|---|---|
| | | | **Bin1** | **Bin2** | **Bin3** | **Bin4** | **Bin5** | **Bin6** | **Bin7** | **Bin8** | **Bin9** | **Bin10** | **Bin11** | **Bin12** | **Bin13** | **Bin14** | **Bin15** | **Bin16** | **Bin17** |
| **Mon** | 8 | 1 | 346537 | 104064 | 25882 | 5852 | 2357 | 1374 | 716 | 657 | 525 | 377 | 312 | 227 | 120 | 55 | 37 | 20 | 14 |
| | | 2 | 463982 | 147575 | 33597 | 6788 | 2419 | 1348 | 685 | 612 | 422 | 289 | 191 | 175 | 95 | 40 | 30 | 15 | 13 |
| | | 3 | 364197 | 109392 | 26949 | 5700 | 2131 | 1191 | 610 | 535 | 400 | 274 | 189 | 147 | 90 | 40 | 21 | 6 | 17 |
| | | 4 | 304151 | 87562 | 20028 | 4458 | 2099 | 1320 | 706 | 567 | 405 | 263 | 191 | 130 | 74 | 29 | 21 | 9 | 11 |
| | | 5 | 312049 | 88103 | 19177 | 3817 | 1486 | 704 | 350 | 270 | 187 | 111 | 94 | 56 | 26 | 10 | 8 | 1 | 10 |
| | 12 | 1 | 192279 | 55306 | 12620 | 2742 | 1250 | 863 | 521 | 497 | 393 | 236 | 177 | 142 | 74 | 35 | 29 | 20 | 15 |
| | | 2 | 197087 | 58208 | 14098 | 3320 | 1683 | 1116 | 618 | 538 | 361 | 216 | 161 | 94 | 43 | 24 | 4 | 6 | 10 |
| | | 3 | 188291 | 53549 | 11978 | 2811 | 1387 | 1028 | 540 | 598 | 406 | 247 | 167 | 131 | 65 | 19 | 9 | 5 | 7 |
| | | 4 | 290080 | 67149 | 14645 | 3030 | 1337 | 949 | 538 | 510 | 368 | 254 | 160 | 94 | 49 | 24 | 7 | 5 | 4 |
| | | 5 | 190286 | 54468 | 12251 | 2642 | 1116 | 888 | 472 | 477 | 323 | 234 | 156 | 112 | 65 | 33 | 18 | 7 | 16 |
| | 16 | 1 | 252391 | 71682 | 15401 | 3076 | 1507 | 1238 | 688 | 675 | 546 | 391 | 308 | 270 | 165 | 68 | 47 | 26 | 22 |
| | | 2 | 161219 | 47588 | 13840 | 4729 | 3328 | 2509 | 1580 | 1658 | 807 | 347 | 219 | 117 | 55 | 22 | 7 | 6 | 11 |
| | | 3 | 167756 | 45285 | 9402 | 2025 | 849 | 616 | 268 | 238 | 108 | 68 | 37 | 20 | 5 | 2 | 1 | 0 | 1 |
| | | 4 | 276305 | 75160 | 16209 | 3190 | 1512 | 1053 | 544 | 507 | 349 | 210 | 138 | 85 | 55 | 17 | 10 | 6 | 5 |
| | | 5 | 314877 | 90037 | 19361 | 3603 | 1490 | 1061 | 576 | 480 | 339 | 209 | 152 | 75 | 52 | 12 | 14 | 6 | 6 |
| | 20 | 1 | 489338 | 159332 | 36671 | 6533 | 2370 | 1436 | 758 | 685 | 519 | 318 | 259 | 195 | 103 | 53 | 23 | 16 | 15 |
| | | 2 | 227222 | 70482 | 18373 | 4973 | 2972 | 2205 | 1168 | 1057 | 600 | 291 | 202 | 165 | 75 | 35 | 17 | 16 | 14 |
| | | 3 | 305064 | 89408 | 20982 | 4244 | 2294 | 1676 | 903 | 848 | 604 | 377 | 290 | 206 | 115 | 43 | 36 | 24 | 34 |
| | | 4 | 384516 | 110656 | 23658 | 4148 | 1628 | 1126 | 586 | 486 | 318 | 155 | 103 | 52 | 25 | 5 | 5 | 3 | 4 |
| | | 5 | 466346 | 139334 | 32062 | 5676 | 2460 | 1715 | 882 | 800 | 530 | 358 | 252 | 156 | 82 | 38 | 28 | 19 | 38 |
| **Tue** | 8 | 1 | 518142 | 191573 | 57388 | 10044 | 3445 | 1735 | 797 | 681 | 434 | 272 | 244 | 200 | 136 | 51 | 48 | 25 | 57 |
| | | 2 | 458316 | 166074 | 47792 | 10764 | 4689 | 2760 | 1528 | 1386 | 845 | 406 | 211 | 148 | 82 | 42 | 19 | 23 | 22 |
| | | 3 | 555733 | 203184 | 59988 | 9803 | 3023 | 1442 | 728 | 601 | 337 | 177 | 84 | 56 | 25 | 9 | 9 | 3 | 5 |
| | | 4 | 485336 | 163962 | 41251 | 7933 | 2655 | 1194 | 529 | 325 | 163 | 91 | 36 | 13 | 6 | 2 | 0 | 2 | 0 |
| | | 5 | 457590 | 160072 | 42584 | 8003 | 2773 | 1372 | 764 | 575 | 315 | 187 | 116 | 80 | 31 | 21 | 5 | 6 | 17 |
| | 12 | 1 | 302296 | 100406 | 24600 | 4023 | 1271 | 631 | 343 | 317 | 188 | 120 | 97 | 60 | 35 | 17 | 15 | 14 | 9 |

| | | | | | | | | | | | | | | | | | | | |
|---|---|---|---|---|---|---|---|---|---|---|---|---|---|---|---|---|---|---|---|
| | | 2 | 383525 | 135179 | 39864 | 10676 | 5781 | 3910 | 2051 | 1794 | 862 | 272 | 130 | 73 | 34 | 19 | 5 | 2 | 10 |
| | | 3 | 312981 | 103485 | 25536 | 4093 | 1299 | 682 | 362 | 292 | 221 | 131 | 74 | 57 | 35 | 17 | 14 | 10 | 7 |
| | | 4 | 416657 | 131010 | 31339 | 4934 | 1531 | 858 | 504 | 375 | 255 | 142 | 111 | 60 | 37 | 15 | 11 | 5 | 7 |
| | | 5 | 324098 | 104680 | 25262 | 4221 | 1419 | 888 | 459 | 436 | 299 | 207 | 154 | 94 | 56 | 23 | 19 | 13 | 20 |
| | | 1 | 244734 | 78887 | 17187 | 3074 | 1290 | 779 | 451 | 378 | 328 | 193 | 111 | 121 | 71 | 29 | 15 | 8 | 14 |
| | | 2 | 242760 | 87172 | 23821 | 6789 | 3848 | 2733 | 1447 | 1273 | 649 | 269 | 142 | 92 | 51 | 16 | 11 | 8 | 10 |
| | 16 | 3 | 193704 | 61421 | 12993 | 2486 | 971 | 508 | 277 | 191 | 129 | 64 | 38 | 13 | 12 | 13 | 3 | 1 | 0 |
| | | 4 | 194824 | 58313 | 12476 | 2369 | 1003 | 750 | 417 | 347 | 248 | 127 | 88 | 40 | 10 | 5 | 3 | 2 | 0 |
| | | 5 | 208273 | 66683 | 15449 | 3287 | 1460 | 1049 | 551 | 499 | 392 | 188 | 141 | 86 | 31 | 20 | 16 | 7 | 5 |
| | | 1 | 126529 | 42945 | 11206 | 2934 | 1571 | 1125 | 642 | 690 | 456 | 279 | 241 | 186 | 89 | 41 | 25 | 16 | 15 |
| | | 2 | 199431 | 91687 | 48858 | 20428 | 14099 | 9991 | 5316 | 5328 | 2401 | 882 | 397 | 204 | 74 | 26 | 18 | 11 | 16 |
| | 20 | 3 | 129368 | 39833 | 9734 | 2402 | 1243 | 947 | 520 | 496 | 330 | 185 | 147 | 95 | 45 | 28 | 13 | 4 | 9 |
| | | 4 | 156920 | 45867 | 11457 | 2817 | 1444 | 1149 | 619 | 523 | 299 | 139 | 103 | 58 | 36 | 13 | 6 | 5 | 2 |
| | | 5 | 141108 | 48666 | 16629 | 5514 | 3596 | 2316 | 1179 | 1002 | 457 | 257 | 171 | 125 | 77 | 42 | 24 | 18 | 32 |
| | | 1 | 347272 | 113538 | 30337 | 7820 | 3513 | 2206 | 1074 | 995 | 811 | 569 | 384 | 273 | 129 | 60 | 35 | 16 | 21 |
| | | 2 | 289018 | 102914 | 31852 | 9183 | 4550 | 2486 | 1030 | 868 | 471 | 262 | 180 | 125 | 53 | 15 | 6 | 5 | 3 |
| | 8 | 3 | 364479 | 118205 | 30748 | 7824 | 3561 | 2076 | 1088 | 1048 | 727 | 449 | 328 | 207 | 86 | 40 | 22 | 8 | 19 |
| | | 4 | 382598 | 126242 | 31988 | 7859 | 3170 | 1736 | 891 | 722 | 553 | 366 | 290 | 139 | 68 | 20 | 5 | 5 | 3 |
| | | 5 | 338432 | 107981 | 27821 | 7165 | 3288 | 2035 | 1057 | 919 | 694 | 466 | 317 | 186 | 94 | 29 | 21 | 17 | 7 |
| | | 1 | 300386 | 93912 | 21065 | 4732 | 1940 | 1252 | 634 | 580 | 391 | 273 | 162 | 124 | 66 | 29 | 11 | 11 | 10 |
| | | 2 | 268322 | 79765 | 18122 | 4163 | 1991 | 1431 | 767 | 668 | 502 | 293 | 177 | 139 | 65 | 28 | 15 | 18 | 24 |
| | 12 | 3 | 313373 | 96104 | 21278 | 4641 | 1783 | 1250 | 608 | 576 | 368 | 206 | 151 | 92 | 29 | 18 | 7 | 3 | 3 |
| **Wed** | | 4 | 333065 | 100325 | 23183 | 5275 | 2355 | 1536 | 833 | 806 | 558 | 358 | 228 | 138 | 55 | 26 | 13 | 15 | 8 |
| | | 5 | 315721 | 95699 | 21929 | 5146 | 2231 | 1470 | 712 | 549 | 388 | 237 | 168 | 103 | 42 | 26 | 14 | 16 | 18 |
| | | 1 | 200108 | 58464 | 12820 | 2985 | 1549 | 1219 | 717 | 661 | 460 | 258 | 205 | 135 | 65 | 47 | 18 | 21 | 13 |
| | | 2 | 199620 | 66380 | 22621 | 7309 | 4548 | 3317 | 1702 | 1692 | 970 | 474 | 289 | 202 | 96 | 48 | 30 | 17 | 20 |
| | 16 | 3 | 210753 | 60798 | 13061 | 2831 | 1331 | 973 | 472 | 423 | 287 | 162 | 102 | 60 | 39 | 11 | 8 | 7 | 15 |
| | | 4 | 209328 | 60105 | 12948 | 2927 | 1373 | 1020 | 561 | 445 | 317 | 153 | 87 | 76 | 34 | 15 | 13 | 5 | 13 |
| | | 5 | 276614 | 77805 | 18116 | 4185 | 2172 | 1674 | 935 | 760 | 500 | 255 | 154 | 57 | 26 | 8 | 5 | 1 | 1 |
| | 20 | 1 | 311042 | 119827 | 55873 | 21965 | 16007 | 12661 | 7183 | 7285 | 3574 | 1302 | 561 | 212 | 65 | 22 | 13 | 6 | 20 |
| | | 2 | 233287 | 75299 | 19063 | 5690 | 3132 | 2072 | 1178 | 1022 | 613 | 355 | 310 | 244 | 134 | 58 | 40 | 13 | 22 |

| | | | | | | | | | | | | | | | | | | | |
|---|---|---|---|---|---|---|---|---|---|---|---|---|---|---|---|---|---|---|---|
| | | 3 | 164170 | 59430 | 23039 | 8344 | 5333 | 3677 | 1855 | 1702 | 946 | 396 | 184 | 122 | 67 | 19 | 8 | 10 | 8 |
| | | 4 | 198219 | 63281 | 16290 | 5276 | 3125 | 2251 | 1240 | 1117 | 603 | 347 | 238 | 180 | 95 | 42 | 30 | 19 | 26 |
| | | 5 | 222837 | 69243 | 17587 | 5126 | 3042 | 2064 | 1133 | 924 | 559 | 311 | 200 | 101 | 52 | 17 | 5 | 2 | 8 |
| **Thu** | 8 | 1 | 229091 | 72477 | 21142 | 6854 | 4293 | 3142 | 1624 | 1342 | 739 | 415 | 239 | 139 | 79 | 26 | 6 | 10 | 9 |
| | | 2 | 214023 | 69531 | 17582 | 3700 | 1464 | 869 | 442 | 482 | 396 | 324 | 269 | 173 | 87 | 41 | 23 | 14 | 14 |
| | | 3 | 169976 | 55187 | 16018 | 4555 | 2462 | 1428 | 619 | 461 | 177 | 116 | 73 | 56 | 9 | 2 | 9 | 7 | 6 |
| | | 4 | 180689 | 53684 | 12488 | 2652 | 1121 | 629 | 357 | 290 | 274 | 238 | 189 | 130 | 54 | 16 | 11 | 3 | 5 |
| | | 5 | 183768 | 54086 | 12232 | 2453 | 1072 | 606 | 306 | 272 | 210 | 198 | 142 | 74 | 34 | 14 | 7 | 1 | 5 |
| | 12 | 1 | 185739 | 55363 | 12409 | 2590 | 1085 | 662 | 365 | 326 | 324 | 246 | 195 | 122 | 63 | 26 | 10 | 4 | 18 |
| | | 2 | 154123 | 47851 | 11283 | 2473 | 1095 | 772 | 393 | 400 | 295 | 239 | 171 | 122 | 59 | 35 | 21 | 15 | 15 |
| | | 3 | 127673 | 41526 | 11268 | 3220 | 1783 | 1168 | 619 | 513 | 353 | 239 | 196 | 131 | 70 | 27 | 22 | 6 | 27 |
| | | 4 | 144318 | 43991 | 9564 | 2016 | 954 | 623 | 307 | 266 | 155 | 106 | 61 | 32 | 22 | 3 | 4 | 2 | 1 |
| | | 5 | 170329 | 50921 | 11692 | 2461 | 1025 | 690 | 386 | 364 | 279 | 191 | 148 | 100 | 44 | 25 | 14 | 2 | 4 |
| | 16 | 1 | 159960 | 47394 | 10734 | 2246 | 1016 | 678 | 400 | 318 | 283 | 170 | 147 | 83 | 42 | 17 | 8 | 9 | 2 |
| | | 2 | 126310 | 36731 | 7116 | 1490 | 637 | 465 | 304 | 287 | 234 | 217 | 136 | 133 | 78 | 44 | 33 | 31 | 13 |
| | | 3 | 120151 | 37278 | 10849 | 3421 | 2146 | 1641 | 1001 | 977 | 596 | 302 | 224 | 147 | 75 | 24 | 20 | 13 | 13 |
| | | 4 | 115842 | 34078 | 6622 | 1364 | 651 | 370 | 244 | 201 | 123 | 81 | 53 | 32 | 14 | 7 | 8 | 1 | 2 |
| | | 5 | 125716 | 35883 | 7781 | 1724 | 961 | 720 | 431 | 385 | 244 | 164 | 108 | 54 | 24 | 6 | 8 | 4 | 1 |
| | 20 | 1 | 128296 | 36201 | 7737 | 1709 | 872 | 658 | 341 | 308 | 225 | 142 | 121 | 54 | 24 | 10 | 7 | 2 | 2 |
| | | 2 | 136308 | 45018 | 11158 | 2833 | 1303 | 861 | 493 | 448 | 342 | 237 | 185 | 155 | 104 | 51 | 24 | 29 | 28 |
| | | 3 | 137988 | 47389 | 13333 | 4035 | 2232 | 1441 | 767 | 795 | 409 | 217 | 143 | 109 | 62 | 31 | 9 | 8 | 15 |
| | | 4 | 192615 | 68665 | 18844 | 5351 | 2398 | 1116 | 525 | 481 | 325 | 207 | 173 | 151 | 71 | 40 | 22 | 15 | 18 |
| | | 5 | 155821 | 50402 | 13391 | 3615 | 1928 | 1250 | 731 | 692 | 519 | 285 | 234 | 177 | 83 | 34 | 18 | 7 | 18 |
| **Fri** | 8 | 1 | 160898 | 55861 | 17858 | 6537 | 4436 | 3607 | 2217 | 2431 | 1582 | 811 | 489 | 306 | 106 | 47 | 37 | 21 | 31 |
| | | 2 | 181429 | 58542 | 14488 | 3995 | 1772 | 1191 | 705 | 695 | 536 | 335 | 285 | 232 | 135 | 63 | 46 | 33 | 21 |
| | | 3 | 187002 | 60740 | 15036 | 3905 | 1841 | 1143 | 630 | 611 | 462 | 299 | 209 | 130 | 81 | 50 | 32 | 12 | 6 |
| | | 4 | 207694 | 69325 | 16221 | 4255 | 2014 | 1260 | 667 | 795 | 511 | 321 | 258 | 172 | 140 | 58 | 41 | 16 | 16 |
| | | 5 | 183862 | 58248 | 13808 | 3744 | 1788 | 1112 | 545 | 491 | 311 | 186 | 154 | 107 | 55 | 18 | 14 | 5 | 4 |
| | 12 | 1 | 177529 | 56008 | 13476 | 3702 | 1605 | 1104 | 538 | 507 | 353 | 220 | 162 | 102 | 58 | 21 | 13 | 5 | 7 |
| | | 2 | 94524 | 26476 | 5833 | 1783 | 1123 | 871 | 528 | 578 | 404 | 230 | 208 | 181 | 122 | 56 | 46 | 29 | 37 |
| | | 3 | 120311 | 50682 | 26105 | 10653 | 6706 | 4296 | 2337 | 2431 | 1306 | 482 | 243 | 112 | 56 | 20 | 16 | 11 | 22 |

| | | | | | | | | | | | | | | | | | | | |
|---|---|---|---|---|---|---|---|---|---|---|---|---|---|---|---|---|---|---|---|
| | | 4 | 98006 | 27892 | 6350 | 1673 | 947 | 690 | 420 | 448 | 296 | 136 | 136 | 69 | 42 | 20 | 18 | 13 | 4 |
| | | 5 | 136852 | 32006 | 7452 | 2297 | 1313 | 882 | 538 | 519 | 322 | 176 | 146 | 77 | 23 | 11 | 2 | 6 | 1 |
| | | 1 | 114557 | 32897 | 7788 | 2292 | 1290 | 836 | 506 | 455 | 296 | 153 | 129 | 53 | 31 | 19 | 5 | 2 | 5 |
| | | 2 | 85160 | 23685 | 5032 | 1574 | 928 | 670 | 420 | 431 | 296 | 190 | 168 | 125 | 95 | 46 | 34 | 17 | 16 |
| | 16 | 3 | 78287 | 27374 | 10462 | 4109 | 2670 | 1839 | 986 | 888 | 456 | 193 | 117 | 100 | 31 | 10 | 6 | 7 | 4 |
| | | 4 | 85212 | 23674 | 4999 | 1502 | 870 | 627 | 372 | 389 | 238 | 170 | 118 | 100 | 40 | 26 | 11 | 9 | 10 |
| | | 5 | 89138 | 24249 | 5392 | 1651 | 902 | 667 | 425 | 417 | 229 | 162 | 104 | 58 | 27 | 9 | 0 | 4 | 4 |
| | | 1 | 93758 | 25219 | 5537 | 1653 | 907 | 661 | 413 | 464 | 306 | 170 | 97 | 106 | 52 | 23 | 13 | 8 | 4 |
| | | 2 | 128502 | 40663 | 9328 | 2469 | 1402 | 1037 | 614 | 657 | 484 | 324 | 276 | 187 | 162 | 74 | 54 | 39 | 32 |
| | 20 | 3 | 125301 | 41893 | 11917 | 3950 | 2439 | 1703 | 939 | 957 | 610 | 312 | 224 | 166 | 84 | 37 | 26 | 20 | 20 |
| | | 4 | 114897 | 35744 | 8266 | 2470 | 1498 | 1117 | 661 | 674 | 478 | 297 | 262 | 167 | 81 | 35 | 28 | 15 | 17 |
| | | 5 | 108002 | 30742 | 7089 | 2061 | 1107 | 845 | 458 | 409 | 263 | 157 | 110 | 70 | 37 | 14 | 7 | 3 | 4 |

**Cut points (µm): Bin 1: 0.300; Bin 2: 0.374; Bin 3: 0.465;Bin 4: 0.579;Bin 5: 0.721;Bin 6: 0.897;Bin 7: 1.117;Bin 8: 1.391;Bin 9: 1.732;Bin 10: 2.156;Bin 11: 2.685;*
*Bin 12: 3.343;Bin 13: 4.162;Bin 14: 5.182;Bin 15: 6.451;Bin 16: 8.031;Bin 17: 10.000*
