# Supplementary material for: Markers of Chemical and Microbiological Contamination of the Air in the Sport Centers
Source: Molecules. 2023 Apr 18;28(8):3560. doi: 10.3390/molecules28083560 (PMC10144153; doi:10.3390/molecules28083560)
Supplement: Supplementary file 1 [file molecules-28-03560-s001.zip › Table S2.pdf]

Table S2. Volatile compounds identified in the gym.

No.	RI[a]	Compound name	ID[b]	Background	Gym
				Relative amount (% peak area)[c]	Relative amount (% peak area)[c]
1	-	Butane	MS	-	0.16
2	-	Ethanol	MS, RS	0.49	0.64
3	-	Acetone	MS, RS	-	0.32
4	-	2-Propanol	MS, RS	0.12	0.32
5	528	1.3-Pentadiene	MS, RI	-	0.15
6	551	1-Propanol	MS, RI	-	0.05
7	562	2-Butanone	MS, RI	-	0.02
8	592	Acetic acid	MS, RI	0.93	2.02
9	607	Tetrahydrofuran	MS, RI	0.11	0.03
10	609	2-Methyl-1-propanol	MS, RI	-	0.03
11	615	Methylcyclopentane	MS, RI	0.01	0.02
12	634	Benzene	MS, RI	0.04	0.07
13	639	1-Butanol	MS, RI	0.03	0.05
14	654	3-Methyl-2-butanone	MS, RI	-	0.01
15	663	Pentanal	MS, RI	-	0.04
16	672	Acetoin	MS, RI	-	0.02
17	700	Heptane	MS, RI	-	0.02
18	714	Methylcyclohexane	MS, RI	-	0.03
19	716	2-Methyl-4-pentanone	MS, RI	-	0.23
20	720	3-Methyl-1-butanol	MS, RI	0.14	0.23
21	723	2-Methyl-1-butanol	MS, RI	0.04	0.06
22	747	Toluene	MS, RI	0.27	1.20
23	752	1-Pentanol	MS, RI	0.03	0.08
24	773	Hexanal	MS, RI	0.23	0.48
25	797	Furfural	MS, RI	0.16	0.26
26	800	Octane	MS, RI	0.08	0.15
27	843	Ethylbenzene	MS, RI	0.10	0.15
28	852	p-Xylene	MS, RI	0.14	0.32
29	855	Cyclohexanone	MS, RI	0.15	0.42
30	864	4-Methyloctane	MS, RI	0.31	0.29
31	867	2-Heptanone	MS, RI	-	0.06
32	869	Styrene	MS, RI	0.15	0.44
33	874	m-Xylene	MS, RI	0.10	0.15
34	877	Heptanal	MS, RI	0.03	0.08
35	883	cis-1-Ethyl-3-methyl-cyclohexane	MS, RI	-	0.03
36	887	2-Butoxyethanol	MS, RI	-	0.14
37	900	Nonane	MS, RI	-	0.14
38	909	Cumene	MS, RI	-	0.04
39	926	α-Thujene	MS, RI	-	0.05
40	929	Benzaldehyde	MS, RI	0.17	0.34
41	934	α-Pinene	MS, RI	0.37	0.88
42	949	Camphene	MS, RI	-	0.05

43	958	6-Methyl-5-heptene-2-one	MS, RI	-	0.54
44	967	Phenol	MS, RI	91.70	77.17
45	986	2-Pentylfuran	MS, RI	0.03	0.28
46	987	Octanal	MS, RI	0.04	0.27
47	988	β-Myrcene	MS, RI	-	0.07
48	994	1-Methyl-2-propylcyclohexane	MS, RI	-	0.02
49	995	2,2,4,6,6-Pentamethylheptane	MS, RI	0.07	0.19
50	998	Hexyl acetate	MS, RI	0.02	0.08
51	1000	Decane	MS, RI	0.06	0.29
52	1001	3-Carene	MS, RI	0.33	0.77
53	1003	1-Ethyl-2,3-dimethylbenzene	MS, RI	0.19	0.14
54	1011	m-Cymene	MS, RI	-	0.53
55	1018	2-Ethyl-1-hexanol	MS, RI	0.29	1.19
56	1021	Eucalyptol	MS, RI	0.11	0.43
57	1023	D-Limonene	MS, RI	0.97	2.79
58	1031	Butylcyclohexane	MS, RI	-	0.12
59	1051	Decahydronaphthalene	MS, RI	-	0.12
60	1053	γ-Terpinene	MS, RI	-	0.08
61	1062	Dihydromyrcenol	MS, RI	0.05	0.38
62	1062	2-Ethylhexyl formate	MS, RI	0.34	-
63	1081	Terpinolene	MS, RI	-	0.08
64	1085	Nonanal	MS, RI	0.17	0.24
65	1086	Linalool	MS, RI	0.10	0.24
66	1088	Tetrahydrolinalool	MS, RI	-	0.08
67	1100	Undecane	MS, RI	0.06	0.24
68	1122	Camphor	MS, RI	0.03	0.13
69	1133	Isopulegol	MS, RI	0.05	0.32
70	1134	Citronellal	MS, RI	-	0.14
71	1135	Pentylcyclohexane	MS, RI	-	0.11
72	1145	Isoborneol	MS, RI	0.08	0.38
73	1162	Menthol	MS, RI	-	0.04
74	1165	2-(2-Butoxyethoxy)ethanol	MS, RI	-	0.12
75	1184	Verbenone	MS, RI	-	0.01
76	1187	Decanal	MS, RI	0.06	0.03
77	1194	Octyl acetate	MS, RI	0.05	0.13
78	1200	Dodecane	MS, RI	0.05	0.20
79	1217	2,6-Dimethylundecane	MS, RI	-	0.04
80	1279	Isobornyl acetate	MS, RI	-	0.08
81	1362	3-Hydroxy-2,2,4-trimethylpentyl isobutyrate	MS, RI	0.86	1.04
82	1369	Isobornyl acrylate	MS, RI	0.09	0.30
83	1378	Isobornyl propionate	MS, RI	-	0.11
84	1399	β-Elemene	MS, RI	-	0.02
85	1400	Tetradecane	MS, RI	-	0.11

[a] Retention indices (RI) calculated using a homologous series of n-alkanes (C_5-C_{20})

[b] Identification (ID) of volatile compounds was carried out by comparing: mass spectrum (MS) of components with those from NIST/EPA/NIH mass spectra library; Retention indices (RI) of components with those from literature data; mass spectrum with the mass spectrum of the GC reference standards (RS)

[c] expressed as % of total identified peak areas
