# Supplementary material for: Markers of Chemical and Microbiological Contamination of the Air in the Sport Centers
Source: Molecules. 2023 Apr 18;28(8):3560. doi: 10.3390/molecules28083560 (PMC10144153; doi:10.3390/molecules28083560)
Supplement: Supplementary file 1 [file molecules-28-03560-s001.zip › Table S3.pdf]

Table S3. The average number of microorganisms in tested fitness club

Average microorganisms' number, CFU/m3	Type of microorganism	Sampling time	Sampling location	Day of the week					Type of microorganism	Sampling time	Sampling location	Day of the week				
				Mon	Tue	Wed	Thu	Fri				Mon	Tue	Wed	Thu	Fri
	Bacteria	8	1	3,80x10^2	8,43x10^2	6,50x10^2	1,22x10^3	5,20x10^2	Fungi	8	1	1,06x10^4	2,31x10^3	1,26x10^4	4,49x10^3	6,88x10^3
			2	2,30x10^2	1,10x10^3	4,80x10^2	7,73x10^2	3,90x10^2			2	9,17x10^3	1,26x10^3	2,15x10^3	6,87x10^2	4,19x10^3
			3	5,00x10^2	7,53x10^2	1,80x10^2	8,00x10^2	2,80x10^2			3	9,99x10^3	8,07x10^2	2,21x10^4	1,84x10^3	6,89x10^3
			4	3,63x10^2	5,07x10^2	2,73x10^2	3,63x10^2	1,40x10^2			4	1,21x10^4	1,43x10^3	4,28x10^3	8,93x10^2	1,72x10^3
			5	2,17x10^2	3,93x10^2	2,97x10^2	4,60x10^2	9,00x10^1			5	8,36x10^3	1,43x10^3	5,95x10^3	1,09x10^3	1,92x10^3
			Atm	2,33x10^2	8,03x10^2	2,60x10^2	3,90x10^2	3,30x10^2			Atm	1,12x10^4	4,03x10^3	4,21x10^4	1,73x10^4	8,01x10^3
		12	1	3,63x10^2	7,70x10^1	2,07x10^2	3,13x10^2	9,35x10^2		12	1	9,87x10^3	2,95x10^3	3,01x10^3	9,72x10^3	8,88x10^3
			2	3,77x10^2	1,60x10^3	8,70x10^2	1,47x10^3	5,30x10^2			2	9,67x10^2	5,27x10^2	6,20x10^2	4,33x10^2	1,17x10^3
			3	5,00x10^1	1,37x10^2	7,70x10^1	2,90x10^2	1,25x10^2			3	5,49x10^3	2,19x10^3	1,72x10^3	5,33x10^2	3,60x10^3
			4	1,47x10^2	2,57x10^2	3,53x10^2	1,07x10^2	8,00x10^1			4	2,87x10^3	1,46x10^3	1,82x10^3	4,95x10^3	1,50x10^3
			5	4,30x10^2	2,36x10^3	3,10x10^2	1,23x10^2	1,20x10^2			5	4,63x10^3	1,25x10^3	1,14x10^3	4,74x10^3	1,56x10^3
			Atm	2,00x10^2	2,37x10^2	1,87x10^2	2,43x10^2	8,75x10^2			Atm	2,32x10^4	2,18x10^3	8,12x10^3	1,77x10^4	1,04x10^4
		16	1	1,45x10^3	3,10x10^2	3,23x10^2	2,83x10^2	6,90x10^2		16	1	6,53x10^3	2,15x10^3	1,56x10^3	1,12x10^4	4,69x10^3
			2	4,33x10^2	7,57x10^2	3,48x10^3	1,82x10^3	9,00x10^2			2	6,33x10^2	3,27x10^2	2,20x10^2	2,43x10^3	8,30x10^2
			3	1,33x10^2	2,03x10^2	6,60x10^2	3,70x10^1	3,80x10^2			3	4,47x10^2	4,27x10^2	7,13x10^2	1,23x10^3	2,54x10^3
			4	3,03x10^2	2,63x10^2	1,80x10^2	1,47x10^2	1,25x10^2			4	2,81x10^3	5,87x10^2	4,53x10^2	1,40x10^3	1,85x10^3
			5	4,37x10^2	7,13x10^2	9,30x10^2	4,10x10^2	3,65x10^2			5	4,49x10^3	1,03x10^3	7,93x10^2	2,53x10^3	3,16x10^3
			Atm	1,57x10^2	2,67x10^2	4,17x10^2	2,43x10^2	8,15x10^2			Atm	4,27x10^3	7,94x10^3	8,33x10^3	4,86x10^4	1,05x10^4
		20	1	7,90x10^2	1,72x10^3	6,00x10^2	9,00x10^2	7,59x10^3		20	1	7,83x10^3	8,57x10^3	1,83x10^3	7,38x10^3	3,00x10^4
			2	9,27x10^3	1,83x10^3	2,57x10^3	1,44x10^3	3,66x10^3			2	9,00x10^2	8,67x10^2	1,81x10^3	3,07x10^3	7,32x10^3
			3	1,96x10^3	3,58x10^3	1,54x10^3	2,02x10^3	1,87x10^3			3	4,67x10^2	5,27x10^2	1,37x10^3	9,57x10^3	2,48x10^3
			4	3,10x10^2	9,73x10^2	9,37x10^2	8,47x10^2	5,10x10^2			4	2,51x10^3	8,87x10^2	3,01x10^3	2,69x10^3	1,73x10^3
			5	1,94x10^3	5,31x10^3	1,24x10^3	2,86x10^3	1,81x10^4			5	1,37x10^3	3,05x10^3	1,77x10^3	1,66x10^3	1,89x10^3
			Atm	6,07x10^2	8,43x10^2	2,03x10^2	9,73x10^2	8,45x10^2			Atm	1,34x10^4	2,46x10^4	2,65x10^4	1,39x10^4	5,26x10^4
