# Supplementary figures and images for: Markers of Chemical and Microbiological Contamination of the Air in the Sport Centers
Source: Molecules. 2023 Apr 18;28(8):3560. doi: 10.3390/molecules28083560 (PMC10144153; doi:10.3390/molecules28083560)

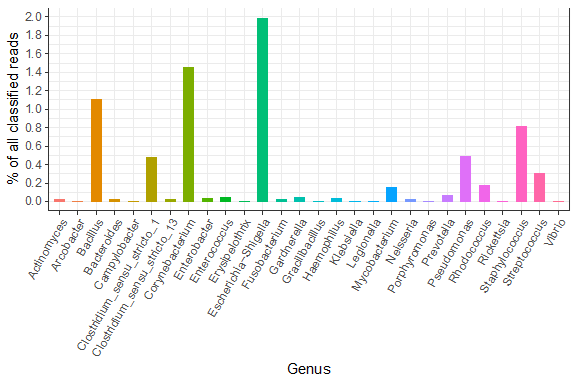

Supplement: Supplementary file 1 [file molecules-28-03560-s001.zip › Figure S3.tif]

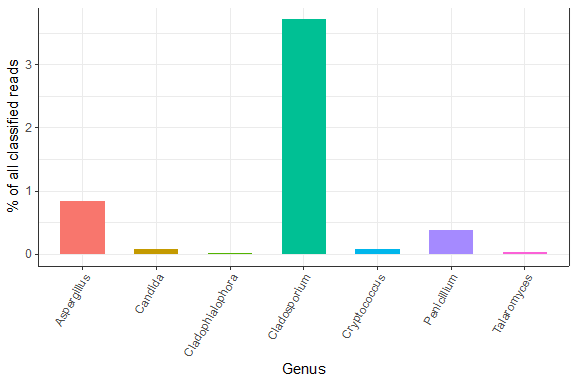

Supplement: Supplementary file 1 [file molecules-28-03560-s001.zip › Figure S4.tif]
